# Predicting Vaccine Effectiveness for Hospitalization and Symptomatic Disease for Novel SARS-CoV-2 Variants Using Neutralizing Antibody Titers

**DOI:** 10.3390/v16030479

**Published:** 2024-03-20

**Authors:** Billy J. Gardner, A. Marm Kilpatrick

**Affiliations:** Department of Ecology and Evolutionary Biology, University of California, Santa Cruz, CA 95060, USA

**Keywords:** vaccine effectiveness, surrogate of protection, SARS-CoV-2, COVID-19, immune evasion, severe disease

## Abstract

The emergence of new virus variants, including the Omicron variant (B.1.1.529) of SARS-CoV-2, can lead to reduced vaccine effectiveness (VE) and the need for new vaccines or vaccine doses if the extent of immune evasion is severe. Neutralizing antibody titers have been shown to be a correlate of protection for SARS-CoV-2 and other pathogens, and could be used to quickly estimate vaccine effectiveness for new variants. However, no model currently exists to provide precise VE estimates for a new variant against severe disease for SARS-CoV-2 using robust datasets from several populations. We developed predictive models for VE against COVID-19 symptomatic disease and hospitalization across a 54-fold range of mean neutralizing antibody titers. For two mRNA vaccines (mRNA-1273, BNT162b2), models fit without Omicron data predicted that infection with the BA.1 Omicron variant increased the risk of hospitalization 2.8–4.4-fold and increased the risk of symptomatic disease 1.7–4.2-fold compared to the Delta variant. Out-of-sample validation showed that model predictions were accurate; all predictions were within 10% of observed VE estimates and fell within the model prediction intervals. Predictive models using neutralizing antibody titers can provide rapid VE estimates, which can inform vaccine booster timing, vaccine design, and vaccine selection for new virus variants.

## 1. Introduction

The evolution of pathogens in populations with high immunity from infection or vaccination can lead to new variants with substantial immune escape [1,2,3,4]. Determining the extent of the effect of immune evasion on the effectiveness of vaccines is critical for assessing the need for new and variant-specific vaccines and determining if additional non-pharmaceutical interventions are needed to limit spread [5]. Traditional vaccine effectiveness (VE) studies can only be performed when there is significant transmission of the new variant in a partially vaccinated population and can be costly. Delays in implementing interventions or developing vaccines until traditional VE studies can be performed could result in rapid growth of a new variant, stress on healthcare systems, and substantial preventable disease and death [5]. Developing and validating faster methods for estimating VE is, therefore, critical [6].

The emergence and rapid spread of the Omicron variant (B.1.1.529) of SARS-CoV-2 in November of 2021 in South African populations raised the possibility that this new variant was highly immune evasive [7]. VE estimates were urgently needed to determine the most effective public health response to the spread of the Omicron variant, which could include third vaccine doses, new vaccines, non-pharmaceutical interventions, or new treatments. Waiting for enough cases to accumulate to estimate VE using observational studies could lead to the rapid spread of the Omicron variant, stress on healthcare systems, and substantial preventable disease and death.

One way to estimate VE against new pathogen variants is to use a surrogate of protection. Protection against infection and severe disease has been correlated with immune responses to several pathogens. Neutralizing antibody titers were correlated with protection against all infections for several arboviruses, and against symptomatic disease for some viruses that replicate in the mucosae [8], including influenza [9]. For some endemic human coronaviruses, IgA in the serum and mucosa was associated with a shorter duration of viral shedding, and neutralizing antibody titers were correlated with protection against symptomatic disease [10]. Establishing correlates of protection can be used for updating vaccines with much smaller immunogenicity-based trials than the randomized control trials needed to establish vaccine efficacy [8,11,12].

Most recently, neutralizing antibody titers have been correlated with protection against COVID-19. Initial studies linked protection against symptomatic disease with neutralizing antibody titers against the original virus that emerged in 2020 [13]. Subsequent work examined VE for both symptomatic and severe disease for three groups of virus variants: pre-Delta, Delta, and Omicron [14]. This study used Spearman’s rank correlations and found a correlation between neutralizing antibody titers and protection against symptomatic and severe disease, but did not fit models for these variants. Instead, it compared vaccine effectiveness estimates to model predictions from an earlier study [13]. Finally, a more recent study fit models using data from England to estimate VE against mild disease, hospitalization, and death for Delta and Omicron [15]. These and other studies [16,17,18,19] provided strong evidence for a correlation between neutralizing antibodies and protection against symptomatic and severe disease, but in all studies, the datasets used to fit the models were limited, either in the range of neutralizing antibody titers examined [13,14,17,18,19], resulting in substantial uncertainty (particularly for models of severe disease [13,14,17]), or in the geographic scale of populations studied [15,16]. All currently circulating SARS-CoV-2 variants are highly immune evasive relative to the initial SARS-CoV-2 variants, making predictions and appropriate uncertainty at low neutralizing antibody titers critical for any prudent use in guiding vaccine decision making [20]. Here, we examine the accuracy of predicting VE for the highly immune-evasive Omicron variant for both mild and severe disease a key vaccine endpoint [21].

Our goal was to build predictive models for VE using neutralizing antibody titers and determine the accuracy of these models in predicting VE for mild and severe disease for the new Omicron virus variant. First, we examined variation in neutralizing antibody titers to determine the extent of data that is needed to accurately measure this surrogate of protection for a new virus variant [22]. Second, we applied a novel statistical approach to examine the relationships between neutralizing antibody titers and VE for two commonly measured and important endpoints, hospitalization and symptomatic disease, using data for seven vaccines and five virus variants, including the immune-evasive Beta variant. Third, we used the relationships to predict VE (including uncertainty) for the Omicron variant for these endpoints. Finally, we assessed the accuracy of these predictions by comparing model predictions with subsequently collected empirical VE estimates. We show that neutralizing antibody titers are an accurate surrogate of protection to quickly estimate VE for both mild and severe disease during a public health emergency.

## 2. Materials and Methods

### 2.1. Relative Neutralizing Antibody Titers by Variant, Vaccine Dose, and with Waning

Following a similar approach to previous studies [13,14,15,17,23], we calculated neutralizing antibody titer ratios (NATRs) as follows. First, we collected data on mean neutralizing antibody titers for SARS-CoV-2 variants, including wild type (WT) (including D614G), Alpha, Beta, Gamma, Delta, and three Omicron variant sub-lineages (BA.1, BA.2, and BA.4/5) (Table S1). We note that neutralizing antibody titers in different labs were measured with different sera, which adds uncertainty to the estimate of mean neutralizing antibody titers. A collection of sera standards would reduce this source of variation. For each vaccine in a study, we normalized mean neutralizing antibody titers for WT virus, T_vac,WT_, by dividing them by the mean neutralizing antibody titers of convalescent sera from that study, T_conv,WT_, to produce a mean neutralizing antibody titer ratio, NATR_vac_ [13]:

NATR_vac_ = (T_vac,WT_/T_conv,WT_) (1)

This produces a measure of the ability of a vaccinated person’s blood to neutralize the wild-type virus relative to the blood of a person that has been infected with wild-type virus. For example, vaccination with BNT162b2, made by Pfizer-BioNTech, resulted in mean neutralizing antibody titers for wild-type (WT) SARS-CoV-2 that were approximately 2.37-fold higher than convalescent sera, producing a NATR_vac_ of 2.37 [13]. For each non-WT variant, we divided the mean neutralizing antibody titer for a vaccine, T_vac,var_, for that variant by the mean neutralizing antibody titer for that vaccine for wild-type (WT) SARS-CoV-2 (including Wuhan-Hu-1, US-WA1/2020, B, and B.1 lineages), T_vac,WT_. This produced a ratio representing the difference between a virus variant and WT virus, NATR_var_:

NATR_var_ = (T_vac,var_/T_vac,WT_) (2)

1/NATRvar is often expressed as the X-fold difference in neutralizing antibody titers. We examined mean neutralizing antibody titers and VE shortly after initial vaccination and for two additional immune statuses for mRNA vaccines: waned two-dose projection (>6 months post 2nd dose) and recently boosted (2–4 weeks) after a third vaccine dose. We used the relative neutralizing antibody titer ratio for these two immune statuses relative to recent two-dose neutralizing antibody titers to produce a third neutralizing antibody titer ratio:

NATR_istatus_ = (T_istatus,vac,WT_/T_vac,two-dose,WT_) (3)

NATR_waned_ was similar for both mRNA vaccines: 0.124 (95% CI: 0.088–0.138) for BNT162b2 and 0.118 (95% CI: 0.115–0.120) for mRNA-1273, indicating that mean neutralizing antibody titers were approximately 8-fold lower 6+ months after vaccination. NATR_boosted_ was 1.54 (95% CI: 1.52–1.57) for mRNA-1273 and 3.22 (95% CI: 2.82–4.24) for BNT162b2 [24]. The total relative neutralizing antibody titer ratio, NATR_tot_, for each vaccine, virus variant, and immune status is

NATR_tot_ = NATR_vac_ × NATR_var_ × NATR_istatus_ (4)

The first term in Equation (4) is the ratio previously used as surrogates of protection for symptomatic disease for COVID-19 from WT virus [13] and the second term is the inverse of the “X-fold reduction” in the neutralization of a non-WT variant relative to WT. The third term is the fold change for a given immune status relative to titers immediately following a second vaccine dose.

In estimating NATR_var_, we only included studies that measured titers for WT virus and at least 2 other virus variants to reduce between-lab differences in titers for single variants (see below). We examined differences among virus variants using a linear mixed effects model with variant and vaccine as fixed effects and study ID as a random effect using the *lme* function in the *lme4* package using R, v4.2.2.

### 2.2. VE against Symptomatic Disease and Hospitalization

We collected VE estimates for COVID-19 from the literature (including a systematic living review [25]) and categorized each study by vaccine, variant type (Alpha, Beta, Gamma, Delta, BA.1, BA.2), and endpoint (hospitalization and symptomatic disease). We excluded estimates where the virus variant for the VE estimate could not be determined.

To fit a relationship between VE and NATR, we needed estimates of the number of infections and population sizes in the vaccine and control groups. However, most studies did not report the number of infections or population sizes; however, all reported a VE and 95% CI. We estimated the effective number of infected individuals in the control group (I_c_) and the effective number of infected individuals in the vaccine group (I_v_) for each study by determining the number of each needed to match the mean and 95% CI given in a study. We held the population of individuals in the control group (N_c_) and vaccine group (N_v_) constant at 1,000,000 because the 95% CI was invariant to variation in these values for observed incidence values. We used a maximum value of 1000 infections in the control group (I_c_) to reduce the undue leverage of some very large studies [26] (Figure S4). We noted that uncertainty was larger (95% CIs were wider) for less common global variants (Beta, Gamma) or less commonly used vaccines (e.g., Johnson and Johnson/Jannsen’s Ad26.COV2.S, Sinovac).

### 2.3. Relationships between VE and Neutralizing Antibody Titers by Vaccine and Variant

We modeled the relationship between NATR_tot_ and VE for each endpoint across all vaccines and variants as

(5) $$VE=1-\frac{1}{1+e^{-c_{0}-c_{1}{log}_{2}\left({NATR}_{tot}\right)}}$$

This mathematical form constrains the mean VE estimate to be between 0 and 1 because we believed it was unlikely that VE would be negative even for very low neutralizing antibody titers. We estimated c_0_ and c_1_ separately for the two endpoints, symptomatic disease and hospitalization, by maximizing the likelihood of observing the number of infections in the control group, I_c_, and vaccine group, I_v_, for each study, where the likelihood was a product of two binomial distributions:

L(N_c_, I_c_, N_v_, I_v_|NATR, b_s_, c_0_, c_1_) = Bi(N_c_, I_c_, b_s_) × Bi(N_v_, I_v_, b_s_ × VE) (6)

Here, b_s_ is the baseline risk for the study period, which is the fraction of control individuals infected during the study. We fit the model in *R* using the *mle2* function in the *bbmle* package.

We used the fitted relationships between VE and neutralizing antibody titer to estimate VE for the Omicron subvariant BA.1 for the two endpoints, symptomatic disease and hospitalization. We report two VE predictions for the BA.1 subvariant of Omicron using two values of NATR_var_. When we first posted a preprint on 11 December 2021, we used a 39-fold reduction in neutralizing antibody titers (NATR_var_ = 1/39), based on the first unpublished data available on 8 December 2021 [27,28]. Subsequent studies suggested a lower value (NATR_var_ = 1/18.5); we also show predictions using this value. We calculated confidence intervals for predictions of the fitted model using the quantiles and covariance of the distributions of the coefficients c_0_ and c_1_ in Equation (5). We calculated prediction intervals using bootstrapping of 10,000 draws using Equation (6) and the fitted models with 10,000 people in both the control and vaccination groups and a baseline risk, b_s_, of 0.0115, which resulted in a mean of 115 infections in the unvaccinated control group, which was similar to the median number of cases in studies we used to fit the models (Tables S4 and S5).

### 2.4. Validating Predictions

We collected data from the literature on VE estimates for the Omicron subvariant BA.1: for two vaccines (mRNA-1273 and BNT162b2), two immune statuses (for people recently vaccinated with a third dose and those with waned immunity, >6 months after two doses), and two endpoints, symptomatic disease and hospitalization. We compared our VE predictions to the validation data; when there were multiple estimates for one of the eight possibilities, we calculated a weighted mean using the inverses of the variance as weights.

All code and data to replicate the results can be found at https://github.com/marmkilpatrick/New-Variant-VE (accessed on 13 March 2024).

## 3. Results

**Figure 1:**
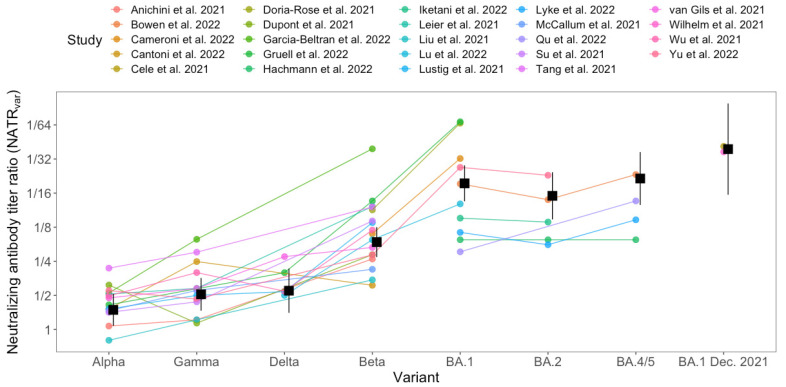
Neutralizing antibody titer ratios for virus variants relative to WT virus (NATR_var_) for five SARS-CoV-2 variants and three subvariants. Each point shows the reduction in neutralizing antibody titer relative to WT virus for a specific vaccine against a virus variant (Table S1). Colors and lines connect points from the same study. Black squares and error bars show the mean and 95% confidence interval for each variant from the fitted model.

There was substantial variation in the NATR_var_ measures for a single virus variant (5–10-fold), but relative variation in NATR_var_ measures across variants was much smaller (Figure 1; Table S1), with clear differences among most variants (Tables S2 and S3). For example, while there was a 4–5-fold range in NATR_var_ measures for each variant, the rank order and relative differences in NATR_var_ measures between variants were quite consistent (Figure 1). Of the variants we considered, Alpha was the least immune evasive, with only a 1.5-fold reduction in neutralizing antibody titers relative to WT (NATR_var_ = 0.64; 95% CI: 0.46–0.88; Tables S2 and S3), and Beta was the most immune evasive before the Omicron variant arose (NATR_var_ = 0.16; 95% CI: 0.12–0.22) (Figure 1; Tables S2 and S3). Initial neutralizing antibody data for the Omicron variant (BA.1) from mid-December 2021 suggested a 39-fold reduction in neutralizing antibody titers (NATR_var_ = 0.026; 95% CI: 0.010–0.063) (Figure 1 rightmost points), whereas subsequent data indicated lower immune evasion (18.5-fold or NATR_var_ = 0.054; 95% CI: 0.050–0.073) (Figure 1).

**Figure 2:**
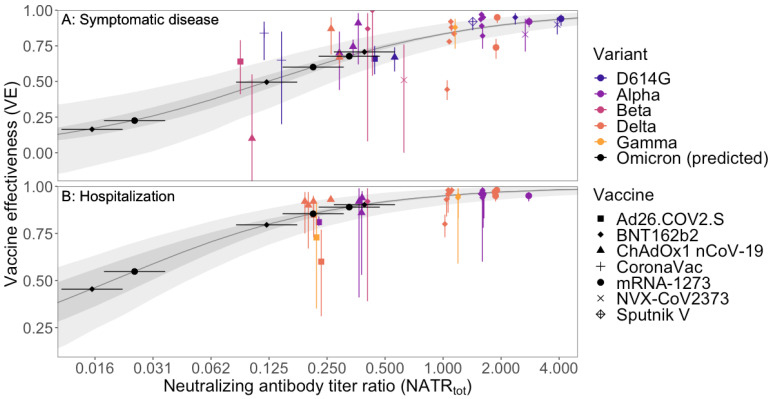
Vaccine effectiveness (VE) plotted against variant- and vaccine-specific neutralizing antibody titer ratios (NATR_tot_) for (**A**) symptomatic disease and (**B**) hospitalization. Each point (and 95% CI), except those for Omicron predictions, represents a single empirical estimate of VE for a single vaccine and virus variant (Tables S4 and S5). Points are jittered slightly along the x-axis to facilitate presentation. Black points show predicted values for the Omicron variant using the fitted model (Table S6); horizontal error bars show 95% CIs for Omicron-specific neutralizing antibody titer ratios from Figure 1. Dark ribbons show 95% CIs for the fitted model, and lighter ribbons show 95% prediction intervals with a mean of 115 infections in the unvaccinated control group (see Section 2).

**Figure 3:**
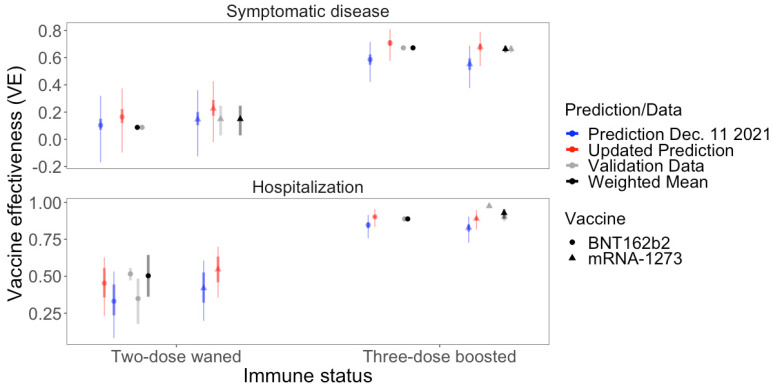
Predicted vaccine effectiveness (thick error bars are 95% CIs; thinner error bars are 95% PIs), VE, for the Omicron variant (BA.1) and observed validation data (and 95% CI), including weighted means, for eight VEs: two endpoints, two vaccines, and two immunity statuses (two-dose waned and three-dose boosted) (Tables S7 and S8). We show VE predictions for both initial NATR_var_ estimates available 8 December 2021 (1/39) (blue) and using subsequent data (NATR_var_ = 1/18.5) (red).

There were strong relationships between VE and NATR_tot_ for both symptomatic disease and hospitalization (Figure 2; Table S6), which enabled us to estimate VE for new virus variants for multiple immune statuses, including waned immunity (Figure 3). These strong relationships were due, in part, to the 54- and 11-fold ranges in neutralizing antibody titers for symptomatic disease and hospitalization, respectively, that existed when we used data from all variants and vaccines together (Figure 2). In contrast, for most individual vaccine-endpoint combinations, data were sparse and there were weak relationships between VE and NATR_tot_ (Figures S1 and S2).

We used the relationships between VE and NATR_tot_ (Figure 2) to predict VE for two mRNA vaccines, BNT162b2 and mRNA-1273, for the BA.1 subvariant of Omicron for waned (two-dose) immunity and after a third-dose booster (Figure 2 and Figure 3; Table S7). We posted a preprint with these VE estimates on 11 December 2021 [29], just 3 days after the first neutralizing antibody titer ratio (NATR_var_) estimates for Omicron became available (8 December 2021).

The fitted model predicted that VE against hospitalization for the Omicron variant would be much lower than for the Delta variant, with the relative risk of hospitalization (1-VE) increasing 2.8–4.4-fold for BNT162b2 and mRNA-1273 for both waned immunity and after a third-dose booster (Figure 2 and Figure 3; Table S7). For example, VE for waned immunity, which comprised the majority of vaccinees in many developed countries in late 2021 [7,24,30,31], against hospitalization for BNT162b2 for the Delta variant was 80.6% (95% CI: 77.5–83.3) but the fitted model predicted that it would be only 46.5% (95% CI: 37.1–56.3) for the Omicron variant, increasing relative risk 2.75-fold (19.4% to 53.5%) (Figure 2 and Figure 3, Table S7). However, the fitted model predicted that a third dose would reverse the loss in protection and increase VE to 90.7% (95% CI: 90.0–91.5) against the Omicron variant (Figure 2 and Figure 3; Table S7). The reductions in VE for Omicron and the benefits of a third-dose booster were similar for the mRNA-1273 vaccine (Figure 2 and Figure 3; Table S7).

The fitted model predicted that VE against symptomatic disease would also be much lower for the Omicron variant than for the Delta variant (Figure 2 and Figure 3; Table S7). For individuals with waned immunity, VE for the BNT162b2 vaccine for symptomatic disease against the Delta variant was 51.6% (95% CI: 46.9–56.4), but the fitted model predicted that protection would be nearly eliminated against the Omicron variant (VE 17.5%, 95% CI: 12.8–23.5) (Figure 2, Figure 3 and Figure S3; Table S7). However, as with protection against hospitalization, the fitted model predicted that boosting with a third vaccine dose would restore VE for symptomatic disease (72.1% (95% CI: 70.2–74.1) for BNT162b2, with similar effects for mRNA-1273; Figure 2, Figure 3 and Figure S3; Table S7).

We compared VE predictions using initial estimates of NATR_var_ (1/39) and updated estimates (NATR_var_ = 1/18.5) for the BA.1 subvariant of Omicron to empirical VE estimates from subsequent observational studies for populations with waned immunity and at two weeks after a third dose (Figure 3; Tables S7 and S8). Weighted means of the validation data fell within the 95% PIs for all seven predictions and all absolute VE errors were less than 10% (Figure 3). VE predictions against symptomatic disease were slightly higher than observed data for the four estimates but no bias was clear in VE predictions against hospitalization (Figure 3). Mean absolute error was 3.0% (sd = 0.9%) for hospitalization and 6.4% (sd = 3.0%) for symptomatic disease. Mean absolute errors were similar for the two vaccines, BNT162b2 (4.9%; sd = 2.9%) and mRNA-1273 (5.1%; sd = 3.5%) (Figure 3).

## 4. Discussion

The emergence and rapid growth of the Omicron (B.1.1.529) variant of SARS-CoV-2 in South Africa, with numerous known and novel mutations in the spike protein, created an urgent need for a predictive model that could be used to estimate VE against this virus [7]. We used the relationships between NATR_tot_ and VE (Figure 2) and NATR_var_ for Omicron to estimate VE for both symptomatic disease and hospitalization on 11 December 2021, three days after the first estimates of NATR_var_ were made available on Twitter/X [24]. Here, we have validated this predictive modeling approach for both symptomatic disease and, importantly, severe disease, by showing that these estimates were consistent with subsequent empirical estimates based on observational data. In the process, we also identified two key components needed for accurately predicting VE for new virus variants using neutralizing antibody titer ratios.

First, we needed multiple measurements of NATR_var_ using multiple virus variants (not just the novel variant and WT virus). We found that single estimates of NATR_var_ varied by almost an order of magnitude, likely due to different sera and methods used in different studies. This highlights the need for standardized neutralization assays and reference sera [22]. However, relative NATR_var_ among virus variants was much more repeatable (Figure 1). Second, to link VE and NATR_tot_, we needed to combine data from multiple vaccines and virus variants to have a sufficiently wide range of NATR_tot_ to estimate VE for an immune-evasive variant like Omicron, especially for waned immunity (Figure 2). Future studies could use VE and NATR_tot_ for individuals with different immunity statuses (waned, recently vaccinated, or with a different number of doses) to broaden the range of NATR and, thereby, strengthen the relationship between VE and NATR_tot_.

The strong correlations between VE for hospitalization and NATR_tot_ using blood serum might, initially, be surprising given the importance of many different arms of the immune system, including T-cells, in protection against severe disease [8,21,32]. However, serum antibody levels frequently correlate with nasal antibody levels (possibly due to transudation from the blood to nasal mucosa) and nasal antibody levels play a key role in protection against infection [33,34]. If antibodies prevent a person from becoming infected, they are also protected against severe disease. It is likely that the correlation between VE against hospitalization and NATR_tot_ partly results from neutralizing antibodies protecting against infection. However, further work is needed to understand the relationships between antibody titers and infectiousness [24].

This study differs from earlier work in the data used to fit the model that is then used to make predictions. Some previous studies had limited datasets for VE for severe disease, which resulted in large uncertainty intervals for VE estimates at low neutralizing antibody titers [13,14], which is problematic for current virus variants. Other studies used data from a single population, and relationships between VE and neutralizing antibody titers for this population differed substantially from other studies [15]. We included a far larger dataset that encompasses more of the variation in the global population than previous studies. This resulted in lower uncertainty for VE predictions and, likely, will reduce bias in future predictions.

Using neutralizing antibody titers to provide initial estimates of VE is much faster than traditional VE studies (e.g., test-negative designs), and can be done for populations where traditional VE studies are difficult. These initial VE estimates can be used for vaccine selection, public health planning, determining whether to implement non-pharmaceutical interventions, and other aspects of public policy. The high accuracy of predicted VE using neutralizing antibody titers compared to validation data for the BA.1 Omicron variant of SARS-CoV-2 suggests that this approach might also be useful for other emerging pathogens (e.g., novel influenza virus strains). A key challenge is obtaining the requisite data needed to build relationships between VE and a surrogate of protection (e.g., neutralizing antibody titer) across a wide enough range to encompass the novel pathogen phenotype and host population immune status. However, given the continued evolution of SARS-CoV-2, influenza [35,36,37,38,39] and many other viruses, and the increased rate of spillover of novel pathogens into human populations, this is an important area for further research.

## Data Availability

All code and data to replicate the results can be found at https://github.com/marmkilpatrick/New-Variant-VE (accessed on 13 March 2024).

## Supplementary Materials

The following supporting information can be downloaded at https://www.mdpi.com/article/10.3390/v16030479/s1, Figure S1–S5; Table S1–S8. References [27,28,40,41,42,43,44,45,46,47,48,49,50,51,52,53,54,55,56,57,58,59,60,61,62,63,64,65,66,67,68,69,70,71,72,73,74,75,76,77,78,79,80,81,82,83,84,85,86,87,88,89,90,91,92,93] are cited in Supplementary Materials.

## References

[1] Grenfell B.T., Pybus O.G., Gog J.R., Wood J.L.N., Daly J.M., Mumford J.A., Holmes E.C. (2004). Unifying the Epidemiological and Evolutionary Dynamics of Pathogens. Science.

[2] Koelle K., Cobey S., Grenfell B., Pascual M. (2006). Epochal Evolution Shapes the Phylodynamics of Interpandemic Influenza A (H3N2) in Humans. Science.

[3] Saad-Roy C.M., Morris S.E., Metcalf C.J.E., Mina M.J., Baker R.E., Farrar J., Holmes E.C., Pybus O.G., Graham A.L., Levin S.A. (2021). Epidemiological and Evolutionary Considerations of SARS-CoV-2 Vaccine Dosing Regimes. Science.

[4] Wagner C.E., Saad-Roy C.M., Morris S.E., Baker R.E., Mina M.J., Farrar J., Holmes E.C., Pybus O.G., Graham A.L., Emanuel E.J. (2021). Vaccine Nationalism and the Dynamics and Control of SARS-CoV-2. Science.

[5] Sonabend R., Whittles L.K., Imai N., Perez-Guzman P.N., Knock E.S., Rawson T., Gaythorpe K.A.M., Djaafara B.A., Hinsley W., FitzJohn R.G. (2021). Non-Pharmaceutical Interventions, Vaccination, and the SARS-CoV-2 Delta Variant in England: A Mathematical Modelling Study. Lancet.

[6] Khoury D.S., Schlub T.E., Cromer D., Steain M., Fong Y., Gilbert P.B., Subbarao K., Triccas J.A., Kent S.J., Davenport M.P. (2023). Correlates of Protection, Thresholds of Protection, and Immunobridging among Persons with SARS-CoV-2 Infection. Emerg. Infect. Dis..

[7] Pulliam J.R.C., van Schalkwyk C., Govender N., von Gottberg A., Cohen C., Groome M.J., Dushoff J., Mlisana K., Moultrie H. (2022). Increased Risk of SARS-CoV-2 Reinfection Associated with Emergence of Omicron in South Africa. Science.

[8] Plotkin S.A. (2010). Correlates of Protection Induced by Vaccination. Clin. Vaccine Immunol..

[9] Coudeville L., Bailleux F., Riche B., Megas F., Andre P., Ecochard R. (2010). Relationship between Haemagglutination-Inhibiting Antibody Titres and Clinical Protection against Influenza: Development and Application of a Bayesian Random-Effects Model. BMC Med. Res. Methodol..

[10] Huang A.T., Garcia-Carreras B., Hitchings M.D.T., Yang B., Katzelnick L.C., Rattigan S.M., Borgert B.A., Moreno C.A., Solomon B.D., Trimmer-Smith L. (2020). A Systematic Review of Antibody Mediated Immunity to Coronaviruses: Kinetics, Correlates of Protection, and Association with Severity. Nat. Commun..

[11] Krammer F. (2021). Correlates of Protection from SARS-CoV-2 Infection. Lancet.

[12] Liu J., Mao Q., Wu X., He Q., Bian L., Bai Y., Wang Z., Wang Q., Zhang J., Liang Z. (2022). Considerations for the Feasibility of Neutralizing Antibodies as a Surrogate Endpoint for COVID-19 Vaccines. Front. Immunol..

[13] Khoury D.S., Cromer D., Reynaldi A., Schlub T.E., Wheatley A.K., Juno J.A., Subbarao K., Kent S.J., Triccas J.A., Davenport M.P. (2021). Neutralizing Antibody Levels Are Highly Predictive of Immune Protection from Symptomatic SARS-CoV-2 Infection. Nat. Med..

[14] Cromer D., Steain M., Reynaldi A., Schlub T.E., Khan S.R., Sasson S.C., Kent S.J., Khoury D.S., Davenport M.P. (2023). Predicting Vaccine Effectiveness against Severe COVID-19 over Time and against Variants: A Meta-Analysis. Nat. Commun..

[15] Hogan A.B., Doohan P., Wu S.L., Mesa D.O., Toor J., Watson O.J., Winskill P., Charles G., Barnsley G., Riley E.M. (2023). Estimating Long-Term Vaccine Effectiveness against SARS-CoV-2 Variants: A Model-Based Approach. Nat. Commun..

[16] Hogan A.B., Wu S.L., Doohan P., Watson O.J., Winskill P., Charles G., Barnsley G., Riley E.M., Khoury D.S., Ferguson N.M. (2022). The Value of Vaccine Booster Doses to Mitigate the Global Impact of the Omicron SARS-CoV-2 Variant. medRxiv.

[17] Cromer D., Steain M., Reynaldi A., Schlub T.E., Wheatley A.K., Juno J.A., Kent S.J., Triccas J.A., Khoury D.S., Davenport M.P. (2022). Neutralising Antibody Titres as Predictors of Protection against SARS-CoV-2 Variants and the Impact of Boosting: A Meta-Analysis. Lancet Microbe.

[18] Gilbert P.B., Montefiori D.C., McDermott A.B., Fong Y., Benkeser D., Deng W., Zhou H., Houchens C.R., Martins K., Jayashankar L. (2022). Immune Correlates Analysis of the mRNA-1273 COVID-19 Vaccine Efficacy Clinical Trial. Science.

[19] Fong Y., McDermott A.B., Benkeser D., Roels S., Stieh D.J., Vandebosch A., Le Gars M., Van Roey G.A., Houchens C.R., Martins K. (2022). Immune Correlates Analysis of the ENSEMBLE Single Ad26.COV2.S Dose Vaccine Efficacy Clinical Trial. Nat. Microbiol..

[20] Khoury D.S., Docken S.S., Subbarao K., Kent S.J., Davenport M.P., Cromer D. (2023). Predicting the Efficacy of Variant-Modified COVID-19 Vaccine Boosters. Nat. Med..

[21] Krause P.R., Fleming T.R., Peto R., Longini I.M., Figueroa J.P., Sterne J.A.C., Cravioto A., Rees H., Higgins J.P.T., Boutron I. (2021). Considerations in Boosting COVID-19 Vaccine Immune Responses. Lancet.

[22] Khoury D.S., Wheatley A.K., Ramuta M.D., Reynaldi A., Cromer D., Subbarao K., O’Connor D.H., Kent S.J., Davenport M.P. (2020). Measuring Immunity to SARS-CoV-2 Infection: Comparing Assays and Animal Models. Nat. Rev. Immunol..

[23] Hogan A.B., Wu S.L., Toor J., Doohan P., Watson O.J., Winskill P., Charles G., Barnsley G., Riley E.M., Khoury D.S. (2022). Long Term Vaccination Strategies to Mitigate the Global Impact of SARS-CoV-2 Transmission: A Modelling Study. SSRN J..

[24] Gardner B.J., Kilpatrick A.M. (2021). Third Doses of COVID-19 Vaccines Reduce Infection and Transmission of SARS-CoV-2 and Could Prevent Future Surges in Some Populations: A Modeling Study. medRxiv.

[25] Higdon M.M., Wahl B., Jones C.B., Rosen J.G., Truelove S.A., Baidya A., Nande A.A., ShamaeiZadeh P.A., Walter K.K., Feikin D.R. (2022). A Systematic Review of Coronavirus Disease 2019 Vaccine Efficacy and Effectiveness Against Severe Acute Respiratory Syndrome Coronavirus 2 Infection and Disease. Open Forum Infect. Dis..

[26] Lewnard J.A., Patel M.M., Jewell N.P., Verani J.R., Kobayashi M., Tenforde M.W., Dean N.E., Cowling B.J., Lopman B.A. (2021). Theoretical Framework for Retrospective Studies of the Effectiveness of SARS-CoV-2 Vaccines. Epidemiology.

[27] Cele S., Jackson L., Khoury D.S., Khan K., Moyo-Gwete T., Tegally H., San J.E., Cromer D., Scheepers C., Amoako D. (2021). SARS-CoV-2 Omicron Has Extensive but Incomplete Escape of Pfizer BNT162b2 Elicited Neutralization and Requires ACE2 for Infection. medRxiv.

[28] Wilhelm A., Widera M., Grikscheit K., Toptan T., Schenk B., Pallas C., Metzler M., Kohmer N., Hoehl S., Helfritz F.A. (2021). Reduced Neutralization of SARS-CoV-2 Omicron Variant by Vaccine Sera and Monoclonal Antibodies. medRxiv.

[29] Gardner B.J., Kilpatrick A.M. (2021). Estimates of Reduced Vaccine Effectiveness against Hospitalization, Infection, Transmission and Symptomatic Disease of a New SARS-CoV-2 Variant, Omicron (B.1.1.529), Using Neutralizing Antibody Titers. medRxiv.

[30] Achieving 70% COVID-19 Immunization Coverage by Mid-2022. https://www.who.int.

[31] Kilpatrick A.M. (2023). Ecological and Evolutionary Insights About Emerging Infectious Diseases from the COVID-19 Pandemic. Annu. Rev. Ecol. Evol. Syst..

[32] Goldblatt D., Alter G., Crotty S., Plotkin S.A. (2022). Correlates of Protection against SARS-CoV-2 Infection and COVID-19 Disease. Immunol. Rev..

[33] Butler S.E., Crowley A.R., Natarajan H., Xu S., Weiner J.A., Bobak C.A., Mattox D.E., Lee J., Wieland-Alter W., Connor R.I. (2021). Distinct Features and Functions of Systemic and Mucosal Humoral Immunity Among SARS-CoV-2 Convalescent Individuals. Front. Immunol..

[34] Cohen J.A., Stuart R.M., Rosenfeld K., Lyons H., White M., Kerr C.C., Klein D.J., Famulare M. (2021). Quantifying the Role of Naturally- and Vaccine-Derived Neutralizing Antibodies as a Correlate of Protection against COVID-19 Variants. medRxiv.

[35] Smith D.J., Lapedes A.S., De Jong J.C., Bestebroer T.M., Rimmelzwaan G.F., Osterhaus A.D.M.E., Fouchier R.A.M. (2004). Mapping the Antigenic and Genetic Evolution of Influenza Virus. Science.

[36] Kucharski A.J., Lessler J., Cummings D.A.T., Riley S. (2018). Timescales of Influenza A/H3N2 Antibody Dynamics. PLoS Biol..

[37] Kucharski A.J., Lessler J., Read J.M., Zhu H., Jiang C.Q., Guan Y., Cummings D.A.T., Riley S. (2015). Estimating the Life Course of Influenza A(H3N2) Antibody Responses from Cross-Sectional Data. PLoS Biol..

[38] Quandelacy T.M., Cummings D.A.T., Jiang C.Q., Yang B., Kwok K.O., Dai B., Shen R., Read J.M., Zhu H., Guan Y. (2021). Using Serological Measures to Estimate Influenza Incidence in the Presence of Secular Trends in Exposure and Immuno-modulation of Antibody Response. Influenza Other Respir. Viruses.

[39] Lessler J., Riley S., Read J.M., Wang S., Zhu H., Smith G.J.D., Guan Y., Jiang C.Q., Cummings D.A.T. (2012). Evidence for Antigenic Seniority in Influenza A (H3N2) Antibody Responses in Southern China. PLoS Pathog..

[40] Anichini G., Terrosi C., Gori Savellini G., Gandolfo C., Franchi F., Cusi M.G. (2021). Neutralizing Antibody Response of Vaccinees to SARS-CoV-2 Variants. Vaccines.

[41] Bowen J.E., Addetia A., Dang H.V., Stewart C., Brown J.T., Sharkey W.K., Sprouse K.R., Walls A.C., Mazzitelli I.G., Logue J.K. (2022). Omicron Spike Function and Neutralizing Activity Elicited by a Comprehensive Panel of Vaccines. Science.

[42] Cameroni E., Bowen J.E., Rosen L.E., Saliba C., Zepeda S.K., Culap K., Pinto D., VanBlargan L.A., De Marco A., di Iulio J. (2022). Broadly Neutralizing Antibodies Overcome SARS-CoV-2 Omicron Antigenic Shift. Nature.

[43] Cantoni D., Siracusano G., Mayora-Neto M., Pastori C., Fantoni T., Lytras S., Di Genova C., Hughes J., Lopalco L., on behalf of the Ambulatorio Medico San Luca Villanuova Group (2022). Analysis of Antibody Neutralisation Activity against SARS-CoV-2 Variants and Seasonal Human Coronaviruses NL63, HKU1, and 229E Induced by Three Different COVID-19 Vaccine Platforms. Vaccines.

[44] Davis C., Logan N., Tyson G., Orton R., Harvey W.T., Perkins J.S., Mollett G., Blacow R.M., Peacock T.P., The COVID-19 Genomics UK (COG-UK) Consortium (2021). Reduced Neutralisation of the Delta (B.1.617.2) SARS-CoV-2 Variant of Concern Following Vaccination. PLoS Pathog..

[45] Doria-Rose N.A., Shen X., Schmidt S.D., O’Dell S., McDanal C., Feng W., Tong J., Eaton A., Maglinao M., Tang H. (2021). Booster of mRNA-1273 Strengthens SARS-CoV-2 Omicron Neutralization. medRxiv.

[46] Dupont L., Snell L.B., Graham C., Seow J., Merrick B., Lechmere T., Maguire T.J.A., Hallett S.R., Pickering S., Charalampous T. (2021). Neutralizing Antibody Activity in Convalescent Sera from Infection in Humans with SARS-CoV-2 and Variants of Concern. Nat. Microbiol..

[47] Garcia-Beltran W.F., St. Denis K.J., Hoelzemer A., Lam E.C., Nitido A.D., Sheehan M.L., Berrios C., Ofoman O., Chang C.C., Hauser B.M. (2022). mRNA-Based COVID-19 Vaccine Boosters Induce Neutralizing Immunity against SARS-CoV-2 Omicron Variant. Cell.

[48] Gruell H., Vanshylla K., Tober-Lau P., Hillus D., Schommers P., Lehmann C., Kurth F., Sander L.E., Klein F. (2022). mRNA Booster Immunization Elicits Potent Neutralizing Serum Activity against the SARS-CoV-2 Omicron Variant. Nat. Med..

[49] Hachmann N.P., Miller J., Collier A.Y., Barouch D.H. (2022). Neutralization Escape by SARS-CoV-2 Omicron Subvariant BA.4.6. N. Engl. J. Med..

[50] Iketani S., Liu L., Guo Y., Liu L., Chan J.F.-W., Huang Y., Wang M., Luo Y., Yu J., Chu H. (2022). Antibody Evasion Properties of SARS-CoV-2 Omicron Sublineages. Nature.

[51] Leier H.C., Bates T.A., Lyski Z.L., McBride S.K., X. Lee D., Coulter F.J., Goodman J.R., Lu Z., Curlin M.E., Messer W.B. (2021). Previously Infected Vaccinees Broadly Neutralize SARS-CoV-2 Variants. medRxiv.

[52] Liu Y., Liu J., Xia H., Zhang X., Fontes-Garfias C.R., Swanson K.A., Cai H., Sarkar R., Chen W., Cutler M. (2021). Neutralizing Activity of BNT162b2-Elicited Serum. N. Engl. J. Med..

[53] Lu L., Mok B.W.Y., Chen L.L., Chan J.M.C., Tsang O.T.Y., Lam B.H.S., Chuang V.W.M., Chu A.W.H., Chan W.M., Ip J.D. (2022). Neutralization of Severe Acute Respiratory Syndrome Coronavirus 2 Omicron Variant by Sera from BNT162b2 or CoronaVac Vaccine Recipients. Clin. Infect. Dis..

[54] Lustig Y., Zuckerman N., Nemet I., Atari N., Kliker L., Regev-Yochay G., Sapir E., Mor O., Alroy-Preis S., Mendelson E. (2021). Neutralising Capacity against Delta (B.1.617.2) and Other Variants of Concern Following Comirnaty (BNT162b2, BioNTech/Pfizer) Vaccination in Health Care Workers, Israel. Eurosurveillance.

[55] Lyke K.E., Atmar R.L., Islas C.D., Posavad C.M., Szydlo D., Paul Chourdhury R., Deming M.E., Eaton A., Jackson L.A., Branche A.R. (2022). Rapid Decline in Vaccine-Boosted Neutralizing Antibodies against SARS-CoV-2 Omicron Variant. Cell Rep. Med..

[56] McCallum M., Bassi J., De Marco A., Chen A., Walls A.C., Di Iulio J., Tortorici M.A., Navarro M.-J., Silacci-Fregni C., Saliba C. (2021). SARS-CoV-2 Immune Evasion by the B.1.427/B.1.429 Variant of Concern. Science.

[57] Qu P., Faraone J.N., Evans J.P., Zheng Y.-M., Yu L., Ma Q., Carlin C., Lozanski G., Saif L.J., Oltz E.M. (2022). Durability of Booster mRNA Vaccine against SARS-CoV-2 BA.2.12.1, BA.4, and BA.5 Subvariants. N. Engl. J. Med..

[58] Su D., Li X., He C., Huang X., Chen M., Wang Q., Qin W., Liang Y., Xu R., Wu J. (2021). Broad Neutralization against SARS-CoV-2 Variants Induced by a Modified B.1.351 Protein-Based COVID-19 Vaccine Candidate. bioRxiv.

[59] Tang J., Lee Y., Ravichandran S., Grubbs G., Huang C., Stauft C., Wang T., Golding B., Golding H., Khurana S. (2021). Reduced Neutralization of SARS-CoV-2 Variants by Convalescent Plasma and Hyperimmune Intravenous Immunoglobulins for Treatment of COVID-19. bioRxiv.

[60] Van Gils M.J., Lavell A., van der Straten K., Appelman B., Bontjer I., Poniman M., Burger J.A., Oomen M., Bouhuijs J.H., van Vught L.A. (2022). Antibody Responses against SARS-CoV-2 Variants Induced by Four Different SARS-CoV-2 Vaccines in Health Care Workers in the Netherlands: A Prospective Cohort Study. PLoS Med..

[61] Wu K., Werner A.P., Moliva J.I., Koch M., Choi A., Stewart-Jones G.B.E., Bennett H., Boyoglu-Barnum S., Shi W., Graham B.S. (2021). mRNA-1273 Vaccine Induces Neutralizing Antibodies against Spike Mutants from Global SARS-CoV-2 Variants. bioRxiv.

[62] Yu J., Collier A.Y., Rowe M., Mardas F., Ventura J.D., Wan H., Miller J., Powers O., Chung B., Siamatu M. (2022). Neutralization of the SARS-CoV-2 Omicron BA.1 and BA.2 Variants. N. Engl. J. Med..

[63] Yu X., Wei D., Xu W., Liu C., Guo W., Li X., Tan W., Liu L., Zhang X., Qu J. (2022). Neutralizing Activity of BBIBP-CorV Vaccine-Elicited Sera against Beta, Delta and Other SARS-CoV-2 Variants of Concern. Nat. Comm..

[64] Polinski J.M., Weckstein A.R., Batech M., Kabelac C., Kamath T., Harvey R., Jain S., Rassen J.A., Khan N., Schneeweiss S. (2022). Durability of the Single-Dose Ad26.COV2.S Vaccine in the Prevention of COVID-19 Infections and Hospitalizations in the US Before and During the Delta Variant Surge. JAMA Netw. Open.

[65] Skowronski D.M., Setayeshgar S., Febriani Y., Ouakki M., Zou M., Talbot D., Prystajecky N., Tyson J.R., Gilca R., Brousseau N. (2021). Two-Dose SARS-CoV-2 Vaccine Effectiveness with Mixed Schedules and Extended Dosing Intervals: Test-Negative Design Studies from British Columbia and Quebec, Canada. medRxiv.

[66] Andrews N., Tessier E., Stowe J., Gower C., Kirsebom F., Simmons R., Gallagher E., Chand M., Brown K., Ladhani S.N. (2021). Vaccine Effectiveness and Duration of Protection of Comirnaty, Vaxzevria and Spikevax against Mild and Severe COVID-19 in the UK. medRxiv.

[67] Stowe J., Andrews N., Kirsebom F., Ramsay M., Bernal J.L. (2022). Effectiveness of COVID-19 Vaccines against Omicron and Delta Hospitalisation: Test Negative Case-Control Study. medRxiv.

[68] Haas E.J., Angulo F.J., McLaughlin J.M., Anis E., Singer S.R., Khan F., Brooks N., Smaja M., Mircus G., Pan K. (2021). Impact and Effectiveness of mRNA BNT162b2 Vaccine against SARS-CoV-2 Infections and COVID-19 Cases, Hospitalisations, and Deaths Following a Nationwide Vaccination Campaign in Israel: An Observational Study Using National Surveillance Data. Lancet.

[69] Martínez-Baz I., Miqueleiz A., Casado I., Navascués A., Trobajo-Sanmartín C., Burgui C., Guevara M., Ezpeleta C., Castilla J., Working Group for the Study of COVID-19 in Navarra (2021). Effectiveness of COVID-19 Vaccines in Preventing SARS-CoV-2 Infection and Hospitalisation, Navarre, Spain, January to April 2021. Eurosurveillance.

[70] Nasreen S., Chung H., He S., Brown K.A., Gubbay J.B., Buchan S.A., Fell D.B., Austin P.C., Schwartz K.L., Sundaram M.E. (2022). Effectiveness of COVID-19 Vaccines against Symptomatic SARS-CoV-2 Infection and Severe Outcomes with Variants of Concern in Ontario. Nat. Microbiol..

[71] Bajema K.L., Dahl R.M., Prill M.M., Meites E., Rodriguez-Barradas M.C., Marconi V.C., Beenhouwer D.O., Brown S.T., Holodniy M., Lucero-Obusan C. (2021). Effectiveness of COVID-19 mRNA Vaccines Against COVID-19–Associated Hospitalization—Five Veterans Affairs Medical Centers, United States, February 1–August 6, 2021. MMWR Morb. Mortal. Wkly. Rep..

[72] Tartof S.Y., Slezak J.M., Fischer H., Hong V., Ackerson B.K., Ranasinghe O.N., Frankland T.B., Ogun O.A., Zamparo J.M., Gray S. (2021). Effectiveness of mRNA BNT162b2 COVID-19 Vaccine up to 6 Months in a Large Integrated Health System in the USA: A Retrospective Cohort Study. Lancet.

[73] Bruxvoort K.J., Sy L.S., Qian L., Ackerson B.K., Luo Y., Lee G.S., Tian Y., Florea A., Aragones M., Tubert J.E. (2021). Effectiveness of mRNA-1273 against Delta, Mu, and Other Emerging Variants. medRxiv.

[74] Ranzani O.T., dos Santos Leite R., Castilho L.D., Maymone Gonçalves C.C., Resende G., de Melo R.L., Croda J. (2021). Vaccine Effectiveness of Ad26.COV2.S against Symptomatic COVID-19 and Clinical Outcomes in Brazil: A Test-Negative Study Design. medRxiv.

[75] Lopez Bernal J., Andrews N., Gower C., Gallagher E., Simmons R., Thelwall S., Stowe J., Tessier E., Groves N., Dabrera G. (2021). Effectiveness of Covid-19 Vaccines against the B.1.617.2 (Delta) Variant. N. Engl. J. Med..

[76] Heath P.T., Galiza E.P., Baxter D.N., Boffito M., Browne D., Burns F., Chadwick D.R., Clark R., Cosgrove C., Galloway J. (2021). Safety and Efficacy of NVX-CoV2373 Covid-19 Vaccine. N. Engl. J. Med..

[77] Emary K.R.W., Golubchik T., Aley P.K., Ariani C.V., Angus B.J., Bibi S., Blane B., Bonsall D., Cicconi P., Charlton S. (2021). Efficacy of ChAdOx1 nCoV-19 (AZD1222) Vaccine against SARS-CoV-2 VOC 202012/01 (B.1.1.7). SSRN J..

[78] Sadoff J., Gray G., Vandebosch A., Cárdenas V., Shukarev G., Grinsztejn B., Goepfert P.A., Truyers C., Fennema H., Spiessens B. (2021). Safety and Efficacy of Single-Dose Ad26.COV2.S Vaccine against Covid-19. N. Engl. J. Med..

[79] Shinde V., Bhikha S., Hoosain Z., Archary M., Bhorat Q., Fairlie L., Lalloo U., Masilela M.S.L., Moodley D., Hanley S. (2021). Efficacy of NVX-CoV2373 Covid-19 Vaccine against the B.1.351 Variant. N. Engl. J. Med..

[80] Al Kaabi N., Zhang Y., Xia S., Yang Y., Al Qahtani M.M., Abdulrazzaq N., Al Nusair M., Hassany M., Jawad J.S., Abdalla J. (2021). Effect of 2 Inactivated SARS-CoV-2 Vaccines on Symptomatic COVID-19 Infection in Adults: A Randomized Clinical Trial. JAMA.

[81] Voysey M., Clemens S.A.C., Madhi S.A., Weckx L.Y., Folegatti P.M., Aley P.K., Angus B., Baillie V.L., Barnabas S.L., Bhorat Q.E. (2021). Safety and Efficacy of the ChAdOx1 nCoV-19 Vaccine (AZD1222) against SARS-CoV-2: An Interim Analysis of Four Randomised Controlled Trials in Brazil, South Africa, and the UK. The Lancet.

[82] Tanriover M.D., Doğanay H.L., Akova M., Güner H.R., Azap A., Akhan S., Köse Ş., Erdinç F.Ş., Akalın E.H., Tabak Ö.F. (2021). Efficacy and Safety of an Inactivated Whole-Virion SARS-CoV-2 Vaccine (CoronaVac): Interim Results of a Double-Blind, Randomised, Placebo-Controlled, Phase 3 Trial in Turkey. The Lancet.

[83] Soeriaatmadja W. Indonesia Grants Emergency Approval for Sinovac Vaccine. The Straits Times 2021. https://www.straitstimes.com/asia/se-asia/indonesia-grants-emergency-approval-for-sinovac-vaccine.

[84] Baden L.R., El Sahly H.M., Essink B., Kotloff K., Frey S., Novak R., Diemert D., Spector S.A., Rouphael N., Creech C.B. (2021). Efficacy and Safety of the mRNA-1273 SARS-CoV-2 Vaccine. N. Engl. J. Med..

[85] Novavax COVID-19 Vaccine Demonstrates 90% Overall Efficacy and 100% Protection Against Moderate and Severe Disease in PREVENT-19 Phase 3 Trial. https://ir.novavax.com/2021-06-14-Novavax-COVID-19-Vaccine-Demonstrates-90-Overall-Efficacy-and-100-Protection-Against-Moderate-and-Severe-Disease-in-PREVENT-19-Phase-3-Trial.

[86] Polack F.P., Thomas S.J., Kitchin N., Absalon J., Gurtman A., Lockhart S., Perez J.L., Pérez Marc G., Moreira E.D., Zerbini C. (2020). Safety and Efficacy of the BNT162b2 mRNA Covid-19 Vaccine. N. Engl. J. Med..

[87] Logunov D.Y., Dolzhikova I.V., Shcheblyakov D.V., Tukhvatulin A.I., Zubkova O.V., Dzharullaeva A.S., Kovyrshina A.V., Lubenets N.L., Grousova D.M., Erokhova A.S. (2021). Safety and Efficacy of an rAd26 and rAd5 Vector-Based Heterologous Prime-Boost COVID-19 Vaccine: An Interim Analysis of a Randomised Controlled Phase 3 Trial in Russia. Lancet.

[88] Tang P., Hasan M.R., Chemaitelly H., Yassine H.M., Benslimane F.M., Al Khatib H.A., AlMukdad S., Coyle P., Ayoub H.H., Al Kanaani Z. (2021). BNT162b2 and mRNA-1273 COVID-19 Vaccine Effectiveness against the SARS-CoV-2 Delta Variant in Qatar. Nat. Med..

[89] Nordström P., Ballin M., Nordström A. (2021). Effectiveness of Heterologous ChAdOx1 nCoV-19 and mRNA Prime-Boost Vaccination against Symptomatic Covid-19 Infection in Sweden: A Nationwide Cohort Study. Lancet Reg. Health.

[90] Tseng H.F., Ackerson B.K., Luo Y., Sy L.S., Talarico C.A., Tian Y., Bruxvoort K.J., Tubert J.E., Florea A., Ku J.H. (2022). Effectiveness of mRNA-1273 against SARS-CoV-2 Omicron and Delta Variants. Nat. Med..

[91] Hansen C., Schelde A., Moustsen-Helm I., Embor H.-D., Eriksen R., Stegger M., Krause T., Mølbak K., Valentiner-Branth P. (2022). Vaccine Effectiveness against Infection and COVID-19-Associated Hospitalisation with the Omicron (B.1.1.529) Variant after Vaccination with the BNT162b2 or mRNA-1273 Vaccine: A Nationwide Danish Cohort Study. Res. Sq..

[92] (2021). SARS-CoV-2 Variants of Concern and Variants under Investigation.

[93] Andrews N., Stowe J., Kirsebom F., Toffa S., Rickeard T., Gallagher E., Gower C., Kall M., Groves N., O’Connell A.-M. (2022). Covid-19 Vaccine Effectiveness against the Omicron (B.1.1.529) Variant. N. Engl. J. Med..

