# Supplementary material for: Predicting Vaccine Effectiveness for Hospitalization and Symptomatic Disease for Novel SARS-CoV-2 Variants Using Neutralizing Antibody Titers
Source: Viruses. 2024 Mar 20;16(3):479. doi: 10.3390/v16030479 (PMC10975673; doi:10.3390/v16030479)
Supplement: Supplementary file 1 [file viruses-16-00479-s001.zip › viruses-2898351-supplementary.pdf]

Supplemental Material for

Predicting Vaccine Effectiveness for Hospitalization and Symptomatic Disease for Novel SARS-CoV-2 Variants Using Neutralizing Antibody Titers

Billy J. Gardner and A. Marm Kilpatrick

Table S1. Fold reductions or inverse of neutralizing antibody titers relative to wild-type virus ($1/NATR_{var}$). Comparisons that were not made in the Reference are denoted with a "-".

Ref.	Alpha	Gamma	Delta	Beta	BA.1	BA.2	BA.4/5	BA.1 (Dec. 2021)
[1]	1.1	1.2	-	4.2	-	-	-	-
[2]	-	-	-	-	19.3	14	23.3	-
[3]	-	-	-	7	32.3	-	-	-
[4]	1.5	4	-	2.5	-	-	-	-
[5]	-	-	-	-	-	-	-	41.4
[6]	-	-	7.7	-	-	-	-	-
[7]	-	-	-	11.4	66.3	-	-	-
[8]	2.5	1.1	-	4.6	-	-	-	-
[9]	2.1	6.3	-	39.4	-	-	-	-
[10]	1.6	-	3.2	13.7	68.3	-	-	-
[11]	-	-	-	-	6.2	6.2	6.2	-
[12]	-	-	-	-	9.6	8.9	-	-
[13]	2.1	2.3	-	12.2	-	-	-	-
[14]	-.8	1.2	-	2.7	-	-	-	-
[15]	-	-	2	6.2	12.9	-	-	-
[16]	1.5	2	2.2	8.8	-	-	-	-
[17]	-	-	-	-	7.2	5.6	9.3	-
[18]	1.5	2.2	-	3.4	-	-	-	-
[19]	-	-	-	-	4.9	-	13.6	-
[20]	1.4	1.8	-	9.1	-	-	-	-
[21]	3.5	4.8	-	12.1	-	-	-	-
[22]	1.9	2.3	4.4	5.3	-	-	-	-
[23]	-	-	-	-	-	-	-	37

[24]	2	3.2	2.2	7.5	-	-	-	-
[25]	-	-	-	-	27	23	-	-
[26]	2.2	1.9	-	4.6	-	-	-	-

Table S2. The fitted model for the fold-reduction ln(1/NATRvar) in neutralizing antibody titers for virus variants relative to WT with variant as a fixed effect and study as a random effect. Alpha was the reference level; the standard deviation of the random effect, study, was 0.51.

Predictor	Estimate	SE	t-value	P-value
Alpha (Intercept)	0.45	0.16	2.75	0.0087
Gamma	0.32	0.16	2.05	0.048
Delta	0.37	0.23	1.63	0.11
Beta	1.37	0.15	9.26	<0.001
BA.1	2.47	0.22	11.06	<0.001
BA.2	2.17	0.29	7.49	<0.001
BA.4/5	2.54	0.31	8.25	<0.001
BA.1 Dec. 2021	3.22	0.49	6.63	<0.001

Table S3. Estimates of fold-reduction ($1/NATR_{var}$) in neutralizing antibody titers for virus variants relative to WT with 95% CIs.

Variant	Fold Reduction (95% CI)
Alpha	1.57 (1.14-2.16)
Gamma	2.15 (1.55-2.99)
Delta	2.27 (1.45-3.55)
Beta	6.17 (4.58-8.30)
BA.1	18.5 (12.8-26.7)
BA.2	13.6 (8.3-22.4)
BA.4/5	19.9 (11.6-34.1)
BA.1 (Dec. 2021)	39.1 (16.0-96.0)

Table S4. Vaccine effectiveness against symptomatic disease estimates used to fit the VE-NATR$_{tot}$ model and shown in Figure 2A.

Variant	Ref.	Vaccine	VE	95% CI	Effective cases vaccine group	Effective cases control group
Alpha	[31]	BNT162b2	0.97	0.97-0.97	30	1000[a]
Alpha	[32]	BNT162b2	0.82	0.73-0.88	27.9	155.2
Alpha	[33]	BNT162b2	0.89	0.87-0.9	110	1000[b]
Alpha	[33]	mRNA-1273	0.92	0.88-0.95	24.7	308.2
Alpha	[33]	ChAdOx1 nCoV-19	0.91	0.62-0.98	2	22.4
Alpha	[38]	BNT162b2	0.937	0.92-0.95	49.7	788.2
Alpha	[29]	BNT162b2	0.95	0.94-0.96	50	1000[c]
Alpha	[38]	ChAdOx1 nCoV-19	0.745	0.68-0.79	104.5	409.7
Alpha	[39]	NVX-CoV2373	0.83	0.71-0.94	14.1	82.7
Alpha	[40]	ChAdOx1 nCoV-19	0.7	0.44-0.85	12.6	42.1
Beta	[33]	BNT162b2	0.87	0.08-0.98	1.1	8.7
Beta	[33]	BNT162b2	1	0.54-1	0	17.8
Beta	[41]	Ad26.COV2.S	0.64	0.41-0.79	21	58.2
Beta	[42]	NVX-CoV2373	0.51	0-0.76	11.2	22.9
Beta	[43]	ChAdOx1 nCoV-19	0.1	-0.77-0.55	15.7	17.5
D614G	[41]	Ad26.COV2.S	0.66	0.55-0.75	62.2	182.9
D614G	[44]	ChAdOx1 nCoV-19	0.67	0.57-0.74	81.9	248.1
D614G	[45]	CoronaVac	0.84	0.65-0.92	7.5	47
D614G	[46]	CoronaVac	0.65	0.20-0.85	7.4	21.2
D614G	[47]	mRNA-1273	0.94	0.89-0.97	10.7	177.9
D614G	[48]	NVX-CoV2373	0.9	0.83-0.95	14.5	145.4
D614G	[49]	BNT162b2	0.95	0.90-0.98	8.2	164.2
D614G	[50]	Sputnik V	0.92	0.86-0.95	13.9	173.5
Delta	[33]	BNT162b2	0.92	0.90-0.94	69.6	869.9
Delta	[33]	mRNA-1273	0.95	0.91-0.97	11.6	233
Delta	[33]	ChAdOx1 nCoV-19	0.87	0.69-0.95	5.8	44.5
Delta	[38]	BNT162b2	0.88	0.85-0.90	108.3	902.5
Delta	[51]	BNT162b2	0.444	0.37-0.51	381	685

Delta	[29]	BNT162b2	0.835	0.83-0.84	165	1000[d]
Delta	[52]	BNT162b2	0.78	0.78-0.79	220	1000[e]
Delta	[51]	mRNA-1273	0.739	0.66-0.80	67.0	257
Delta	[38]	ChAdOx1 nCoV-19	0.67	0.61-0.72	201.2	609.8
Gamma	[33]	BNT162b2	0.88	0.73-0.94	6.4	53.3

[a-e]Estimated sample sizes in the control group (I_c) and vaccine group (I_v) to match the original study VE 95% CI; these were rescaled to limit I_c to 1000 but maintain the correct VE: [a]I_c = 19,183, I_v = 575; [b] I_c = 1973, I_v = 217; [c] I_c = 1855, I_v = 93; [d] I_c = 270,217, I_v = 44,586; [e] I_c = 40,917, I_v = 9002

Table S5. Vaccine effectiveness against hospitalization estimates used to fit the VE-$NATR_{tot}$ model and shown in Figure 2B.

Variant	Ref.	Vaccine	VE	95% CI	Est. cases vaccine group	Est. cases control group
Alpha	[27]	Ad26.COV2.S	0.81	0.79-0.84	190	1000[a]
Alpha	[28]	BNT162b2	0.96	0.83-0.99	1.9	46.9
Alpha	[29]	BNT162b2	0.979	0.91-1	2	94
Alpha	[29]	ChAdOx1 nCoV-19	0.939	0.85-0.98	5	81.6
Alpha	[30]	BNT162b2	0.95	0.78-0.99	1.8	36.6
Alpha	[30]	ChAdOx1 nCoV-19	0.86	0.53-0.96	3	21.3
Alpha	[31]	BNT162b2	0.972	0.97-0.98	28	1000[b]
Alpha	[32]	BNT162b2	0.94	0.6-0.99	1.1	18.6
Alpha	[33]	BNT162b2	0.96	0.94-0.97	27.3	681.3
Alpha	[33]	mRNA-1273	0.95	0.92-0.97	17.8	356.3
Alpha	[33]	ChAdOx1 nCoV-19	0.92	0.41-0.99	1	12.9
Beta	[33]	BNT162b2	0.92	0.39-0.99	1	12.4
Delta	[27]	Ad26.COV2.S	0.85	0.73-0.91	12.9	85.8
Delta	[34]	Ad26.COV2.S	0.6	0.31-0.77	17.8	44.5
Delta	[34]	BNT162b2	0.8	0.73-0.85	52.2	261
Delta	[34]	mRNA-1273	0.95	0.92-0.97	17.4	347.5
Delta	[28]	BNT162b2	0.98	0.97-0.98	20	1000[c]
Delta	[28]	mRNA-1273	0.97	0.96-0.98	30	1000[d]
Delta	[28]	ChAdOx1 nCoV-19	0.92	0.86-0.95	13.5	169.2

Delta	[29]	BNT162b2	0.967	0.96-0.97	33	1000[e]
Delta	[29]	ChAdOx1 nCoV-19	0.93	0.92-0.94	70	1000[f]
Delta	[35]	BNT162b2	0.93	0.84-0.96	6	85.8
Delta	[36]	BNT162b2	0.976	0.93-0.99	3.3	135.6
Delta	[30]	BNT162b2	0.96	0.86-0.99	2.6	65
Delta	[30]	ChAdOx1 nCoV-19	0.92	0.75-0.97	3	37.7
Delta	[33]	BNT162b2	0.98	0.96-0.99	8.2	410.4
Delta	[33]	mRNA-1273	0.98	0.93-1	2.5	123.6
Delta	[33]	ChAdOx1 nCoV-19	0.9	0.67-0.97	2.9	28.7
Gamma	[28]	BNT162b2	0.95	0.83-0.99	2.7	53.6
Gamma	[37]	Ad26.COV2.S	0.729	0.35-0.91	6.2	23
Gamma	[33]	BNT162b2	0.94	0.59-0.99	1.1	18.3

[a-f]Estimated sample sizes in the control group (I_c) and vaccine group (I_v) to match the original study VE 95% CI; these were rescaled to limit I_c to 1000 but maintain the correct VE: [a]I_c = 1469, I_v = 279; [b] I_c = 8784, I_v = 245; [c] I_c = 1794, I_v = 36; [d] I_c = 1289, I_v = 39; [e] I_c = 10577, I_v = 349; [f] I_c = 9085, I_v = 636

Table S6. Statistics for the fitted model between neutralizing antibody titers and VE given by the equation $\mathrm{VE} = 1 - \frac{1}{1+e^{-c_0-c_1\log_2(\mathrm{NATR_{tot}})}}$.

Endpoint	Coefficient	Estimate	SE	Z value	p-value
Symptomatic Disease	c_0	-1.60	0.028	-56.4	$< 2.2\times10^{-16}$
	c_1	0.53	0.031	-17.2	$< 2.2\times10^{-16}$
Hospitalization	c_0	-2.91	0.052	-56.1	$< 2.2\times10^{-16}$
	c_1	-0.51	0.038	-13.6	$< 2.2\times10^{-16}$

Table S7. Predicted VE, 95% CIs and 95% PIs for two vaccines, two variants, two endpoints, and two immune statuses using the model shown in Figure 2.

Variant	Prediction	Vaccine	Endpoint	Status	VE	95% PI	95% CI
Omicron	Updated	mRNA-1273	Hospitalization	Three-dose boosted	0.9	0.82-0.95	0.89-0.91
Omicron	Updated	BNT162b2	Hospitalization	Three-dose boosted	0.91	0.84-0.96	0.9-0.92
Omicron	Updated	mRNA-1273	Hospitalization	Two-dose waned	0.56	0.37-0.71	0.48-0.64

Omicron	Updated	BNT162b2	Hospitalization	Two-dose waned	0.46	0.25-0.63	0.37-0.56
Omicron	Updated	mRNA-1273	Symptomatic disease	Three-dose boosted	0.69	0.56-0.8	0.67-0.72
Omicron	Updated	BNT162b2	Symptomatic disease	Three-dose boosted	0.72	0.6-0.82	0.7-0.74
Omicron	Updated	mRNA-1273	Symptomatic disease	Two-dose waned	0.24	-0.01-0.43	0.18-0.3
Omicron	Updated	BNT162b2	Symptomatic disease	Two-dose waned	0.18	-0.09-0.38	0.13-0.24
Omicron	Updated	mRNA-1273	All infections	Three-dose boosted	0.69	0.56-0.8	0.66-0.72
Omicron	Updated	BNT162b2	All infections	Three-dose boosted	0.71	0.57-0.81	0.68-0.73
Omicron	Updated	mRNA-1273	All infections	Two-dose waned	0.44	0.21-0.62	0.33-0.55
Omicron	Updated	BNT162b2	All infections	Two-dose waned	0.38	0.14-0.59	0.27-0.52
Omicron	Dec. 11 2021	mRNA-1273	Hospitalization	Three-dose boosted	0.83	0.73-0.91	0.81-0.85
Omicron	Dec. 11 2021	BNT162b2	Hospitalization	Three-dose boosted	0.85	0.76-0.92	0.83-0.87
Omicron	Dec. 11 2021	mRNA-1273	Hospitalization	Two-dose waned	0.42	0.19-0.6	0.33-0.52
Omicron	Dec. 11 2021	BNT162b2	Hospitalization	Two-dose waned	0.33	0.08-0.53	0.24-0.44
Omicron	Dec. 11 2021	mRNA-1273	Symptomatic disease	Three-dose boosted	0.56	0.39-0.7	0.52-0.6
Omicron	Dec. 11 2021	BNT162b2	Symptomatic disease	Three-dose boosted	0.59	0.43-0.72	0.56-0.63
Omicron	Dec. 11 2021	mRNA-1273	Symptomatic disease	Two-dose waned	0.15	-0.11-0.36	0.11-0.21
Omicron	Dec. 11 2021	BNT162b2	Symptomatic disease	Two-dose waned	0.11	-0.17-0.32	0.07-0.16
Omicron	Dec. 11 2021	mRNA-1273	All infections	Three-dose boosted	0.62	0.46-0.75	0.57-0.67
Omicron	Dec. 11 2021	BNT162b2	All infections	Three-dose boosted	0.64	0.49-0.76	0.6-0.68
Omicron	Dec. 11 2021	mRNA-1273	All infections	Two-dose waned	0.36	0.12-0.57	0.24-0.51

Omicron	Dec. 11 2021	BNT162b2	All infections	Two-dose waned	0.31	0.06-0.54	0.2-0.47
Delta	Delta	mRNA-1273	Hospitalization	Three-dose boosted	0.98	0.94-1	0.97-0.98
Delta	Delta	BNT162b2	Hospitalization	Three-dose boosted	0.98	0.95-1	0.97-0.98
Delta	Delta	mRNA-1273	Hospitalization	Two-dose waned	0.86	0.77-0.93	0.84-0.87
Delta	Delta	BNT162b2	Hospitalization	Two-dose waned	0.81	0.7-0.89	0.78-0.83
Delta	Delta	mRNA-1273	Symptomatic disease	Three-dose boosted	0.92	0.86-0.97	0.91-0.93
Delta	Delta	BNT162b2	Symptomatic disease	Three-dose boosted	0.93	0.87-0.97	0.92-0.94
Delta	Delta	mRNA-1273	Symptomatic disease	Two-dose waned	0.61	0.45-0.74	0.58-0.65
Delta	Delta	BNT162b2	Symptomatic disease	Two-dose waned	0.52	0.33-0.66	0.47-0.56
Delta	Delta	mRNA-1273	All infections	Three-dose boosted	0.84	0.74-0.92	0.8-0.88
Delta	Delta	BNT162b2	All infections	Three-dose boosted	0.85	0.75-0.93	0.81-0.89
Delta	Delta	mRNA-1273	All infections	Two-dose waned	0.65	0.5-0.77	0.61-0.69
Delta	Delta	BNT162b2	All infections	Two-dose waned	0.6	0.43-0.73	0.54-0.66

Table S8. VE validation data for the Omicron variant shown in Figure 3 for two vaccines, two endpoints, and two immune statuses (two-dose waned and boosted with a third dose).

Ref.	Vaccine	Dose	Endpoint	VE	CI	Est. cases vaccine group	Est. cases control group
[53]	mRNA-1273	Three-dose boosted	Hospitalization	97.5	96.3 - 98.3	25.9	1037
[54]	BNT162b2	Two-dose waned	Hospitalization	51.6	47.2 - 55.6	776.8	1604.9
[54]	BNT162b2	Three-dose boosted	Hospitalization	88.8	87.3 - 90.1	273.1	2438.4
[54]	mRNA-1273	Three-dose boosted	Hospitalization	90.2	87.3 - 92.5	60.7	618.9

[55]	BNT162b2	Two-dose waned	Hospitalization	34.9	17.7 - 48.4	116.2	178.5
[56]	BNT162b2	Two-dose waned	Symptomatic disease	8.8	7 - 10.5	20033.7	21966.8
[56]	BNT162b2	Three-dose boosted	Symptomatic disease	67.2	66.5 - 67.8	12151	37045.9
[56]	mRNA-1273	Two-dose waned	Symptomatic disease	14.9	2.9 - 24.7	426.3	500.9
[56]	mRNA-1273	Three-dose boosted	Symptomatic disease	66.3	63.7 - 68.8	902.5	2678

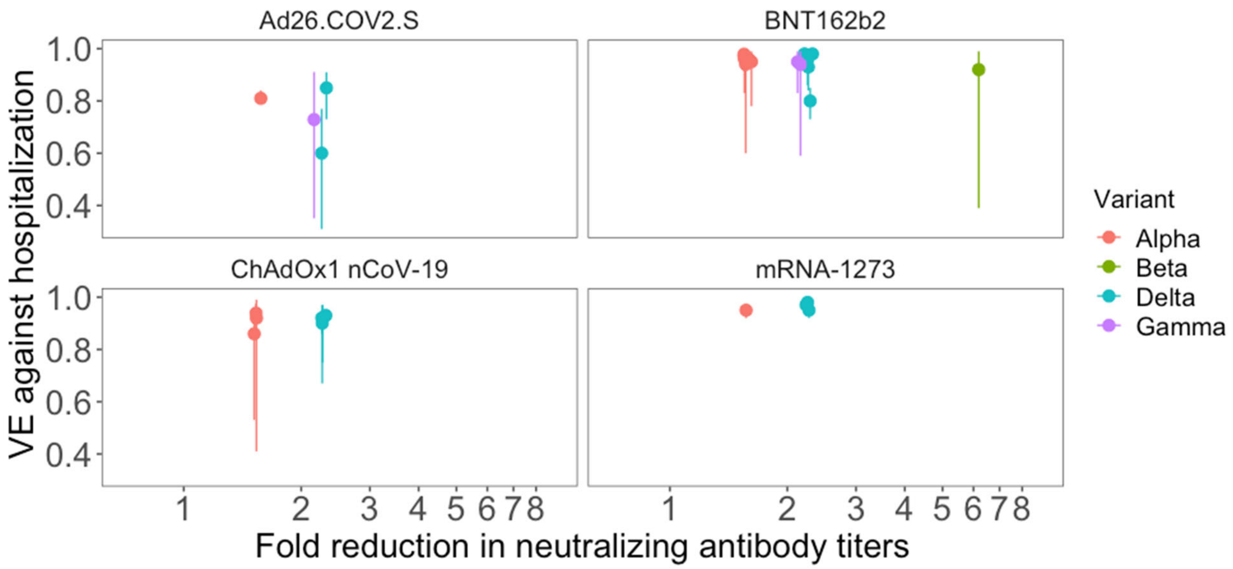

Figure S1. VE against hospitalization by vaccine plotted against variant-specific reductions in neutralizing antibody titers relative to WT virus. Each point (and 95% CI) represents a single estimate of VE for a single vaccine & virus variant from an observational study. Points have been slightly jittered along the x-axis to facilitate presentation.

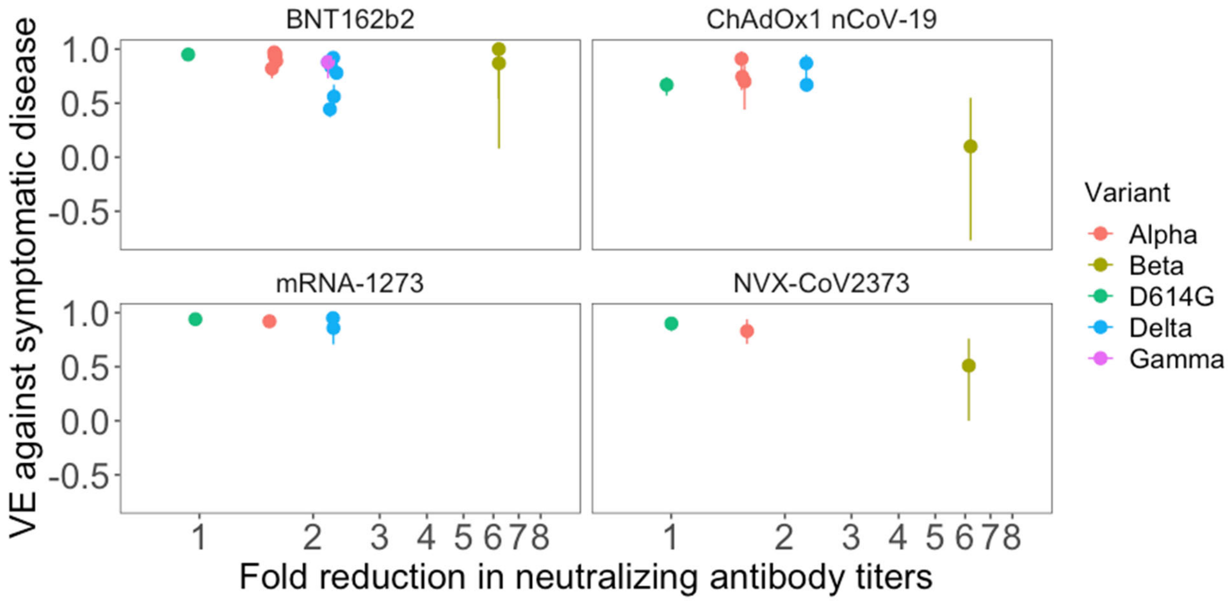

Figure S2. VE against symptomatic disease by vaccine plotted against variant-specific reductions in neutralizing antibody titers relative to WT virus. Each point (and 95% CI) represents a single estimate of VE for a single vaccine & virus variant from an observational study. Points have been slightly jittered along the x-axis to facilitate presentation.

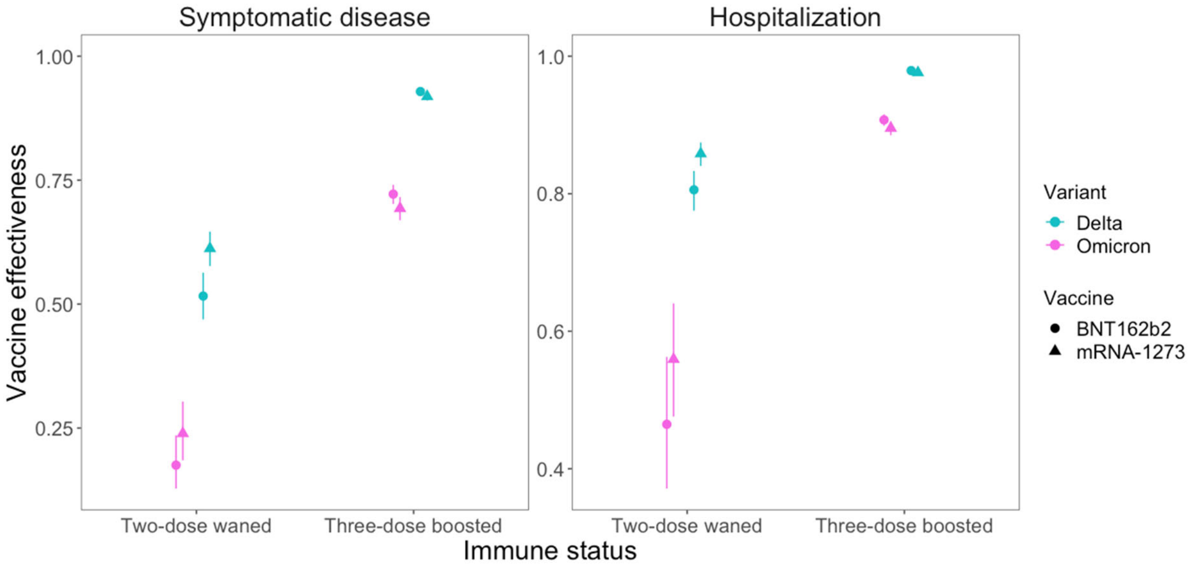

Figure S3. Comparison of estimated VE for the Omicron variant and Delta variants for two endpoints, two vaccines, and two immune statuses (6+ months after two doses (Waned) and shortly after a 3rd dose (Boosted)).

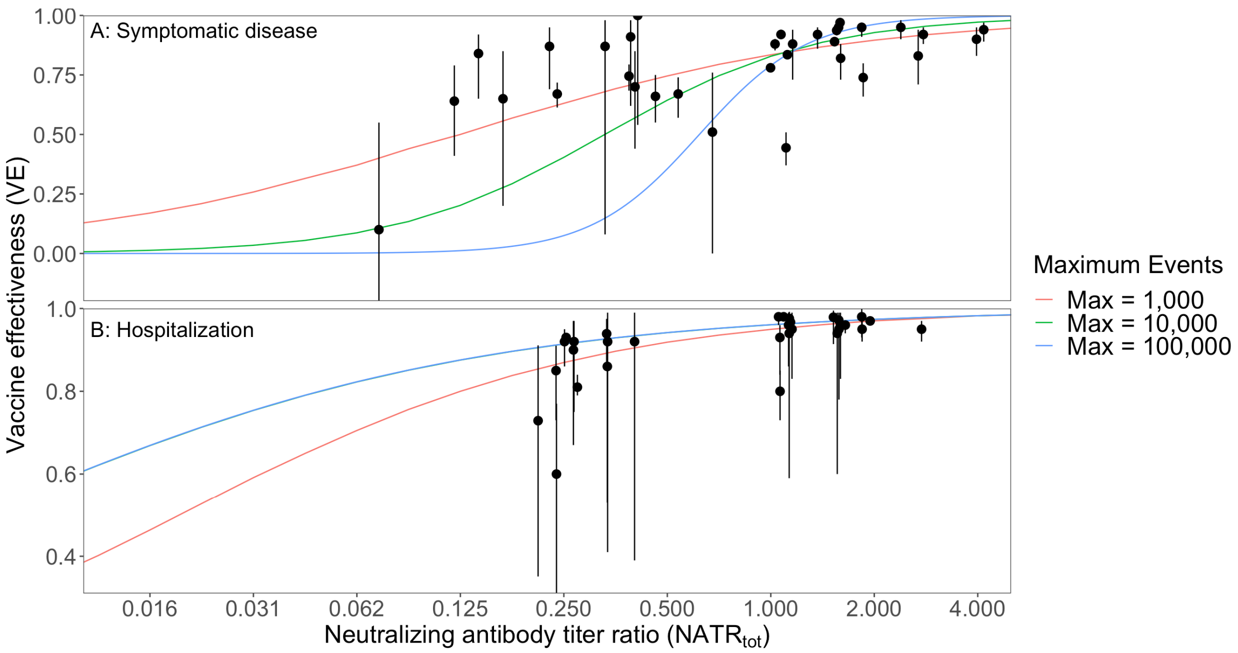

Figure S4. Illustration of the effect of large studies on the fitted relationship between VE and $NATR_{tot}$. Vaccine Effectiveness (VE) is plotted against variant- and vaccine-specific neutralizing antibody titer ratios ($NATR_{tot}$) for (A) symptomatic disease and (B) hospitalization. Each point (and 95% CI represents a single empirical estimate of VE for a single vaccine & virus variant. Points are jittered slightly along the x-axis to facilitate presentation. Each line shows the fitted model using a different maximum value for events (symptomatic cases or hospitalizations) in the control group, corresponding to a different maximum weight given to any one data point. When the maximum value is 10,000 or 100,000, a few points at high $NATR_{tot}$ with very large sample sizes distort the relationship leading to a poor fit at low $NATR_{tot}$.

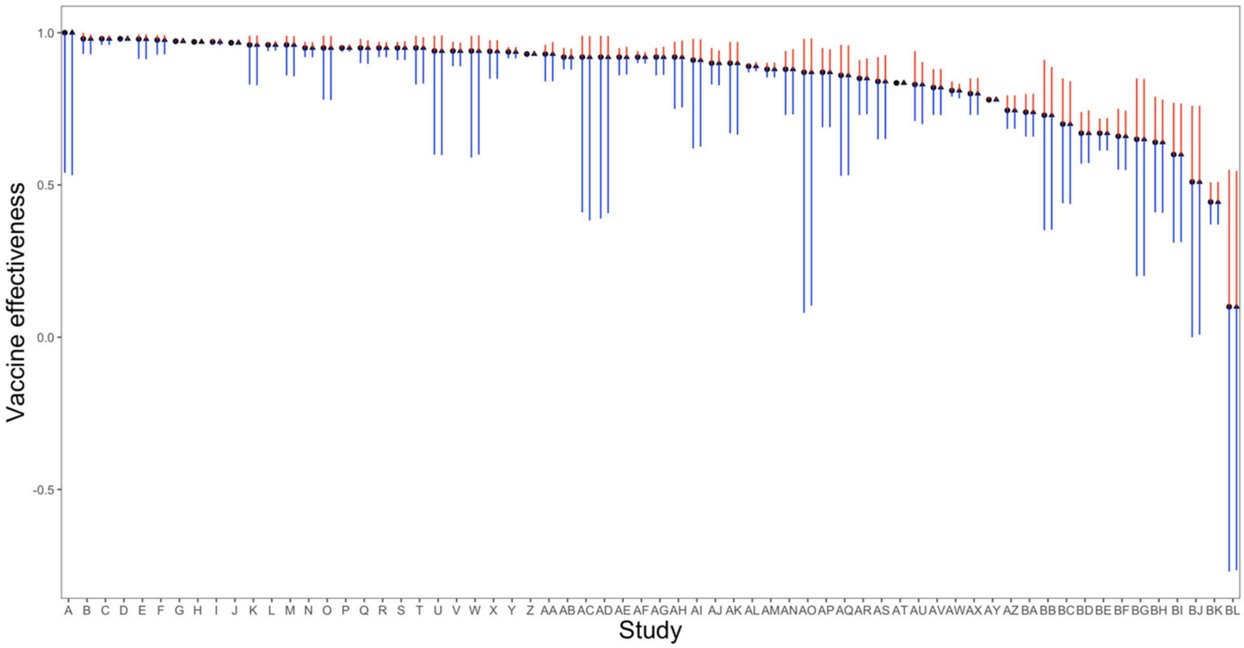

Figure S5. Vaccine effectiveness and 95% CIs for each study (circles) and generated using estimated infections in each arm of each study (triangles), which were not reported in most studies. Upper CIs are shown in red and lower CIs are shown in blue.

References

1. Anichini, G.; Terrosi, C.; Gori Savellini, G.; Gandolfo, C.; Franchi, F.; Cusi, M.G. Neutralizing Antibody Response of Vaccinees to SARS-CoV-2 Variants. *Vaccines* **2021**, *9*, 517, doi:10.3390/vaccines9050517.
2. Bowen, J.E.; Addetia, A.; Dang, H.V.; Stewart, C.; Brown, J.T.; Sharkey, W.K.; Sprouse, K.R.; Walls, A.C.; Mazzitelli, I.G.; Logue, J.K.; et al. Omicron Spike Function and Neutralizing Activity Elicited by a Comprehensive Panel of Vaccines. *Science* **2022**, *377*, 890–894, doi:10.1126/science.abq0203.
3. Cameroni, E.; Bowen, J.E.; Rosen, L.E.; Saliba, C.; Zepeda, S.K.; Culap, K.; Pinto, D.; VanBlargan, L.A.; De Marco, A.; di Iulio, J.; et al. Broadly Neutralizing Antibodies Overcome SARS-CoV-2 Omicron Antigenic Shift. *Nature* **2022**, *602*, 664–670, doi:10.1038/s41586-021-04386-2.
4. Cantoni, D.; Siracusano, G.; Mayora-Neto, M.; Pastori, C.; Fantoni, T.; Lytras, S.; Di Genova, C.; Hughes, J.; on behalf of the Ambulatorio Medico San Luca Villanuova Group; Lopalco, L.; et al. Analysis of Antibody Neutralisation Activity against SARS-CoV-2 Variants and Seasonal Human Coronaviruses NL63, HKU1, and 229E Induced by Three Different COVID-19 Vaccine Platforms. *Vaccines* **2022**, *11*, 58, doi:10.3390/vaccines11010058.
5. Cele, S.; Jackson, L.; Khoury, D.S.; Khan, K.; Moyo-Gwete, T.; Tegally, H.; San, J.E.; Cromer, D.; Scheepers, C.; Amoako, D.; et al. SARS-CoV-2 Omicron Has Extensive but Incomplete Escape of Pfizer BNT162b2 Elicited Neutralization and Requires ACE2 for Infection. *medRxiv* **2021**, doi:10.1101/2021.12.08.21267417.

6. Davis, C.; Logan, N.; Tyson, G.; Orton, R.; Harvey, W.T.; Perkins, J.S.; Mollett, G.; Blacow, R.M.; The COVID-19 Genomics UK (COG-UK) Consortium; Peacock, T.P.; et al. Reduced Neutralisation of the Delta (B.1.617.2) SARS-CoV-2 Variant of Concern Following Vaccination. *PLOS Pathogens* **2021**, *17*, e1010022, doi:10.1371/journal.ppat.1010022.
7. Doria-Rose, N.A.; Shen, X.; Schmidt, S.D.; O'Dell, S.; McDanal, C.; Feng, W.; Tong, J.; Eaton, A.; Maglinao, M.; Tang, H.; et al. Booster of mRNA-1273 Strengthens SARS-CoV-2 Omicron Neutralization. *medRxiv* **2021**, doi:10.1101/2021.12.15.21267805.
8. Dupont, L.; Snell, L.B.; Graham, C.; Seow, J.; Merrick, B.; Lechmere, T.; Maguire, T.J.A.; Hallett, S.R.; Pickering, S.; Charalampous, T.; et al. Neutralizing Antibody Activity in Convalescent Sera from Infection in Humans with SARS-CoV-2 and Variants of Concern. *Nat. Microbiol.* **2021**, *6*, 1433–1442, doi:10.1038/s41564-021-00974-0.
9. Garcia-Beltran, W.F.; St. Denis, K.J.; Hoelzemer, A.; Lam, E.C.; Nitido, A.D.; Sheehan, M.L.; Berrios, C.; Ofoman, O.; Chang, C.C.; Hauser, B.M.; et al. mRNA-Based COVID-19 Vaccine Boosters Induce Neutralizing Immunity against SARS-CoV-2 Omicron Variant. *Cell* **2022**, *185*, 457-466.e4, doi:10.1016/j.cell.2021.12.033.
10. Gruell, H.; Vanshylla, K.; Tober-Lau, P.; Hillus, D.; Schommers, P.; Lehmann, C.; Kurth, F.; Sander, L.E.; Klein, F. mRNA Booster Immunization Elicits Potent Neutralizing Serum Activity against the SARS-CoV-2 Omicron Variant. *Nat. Med.* **2022**, *28*, 477–480, doi:10.1038/s41591-021-01676-0.
11. Hachmann, N.P.; Miller, J.; Collier, A.Y.; Barouch, D.H. Neutralization Escape by SARS-CoV-2 Omicron Subvariant BA.4.6. *N. Engl. J. Med.* **2022**, *387*, 1904–1906, doi:10.1056/NEJMc2212117.
12. Iketani, S.; Liu, L.; Guo, Y.; Liu, L.; Chan, J.F.-W.; Huang, Y.; Wang, M.; Luo, Y.; Yu, J.; Chu, H.; et al. Antibody Evasion Properties of SARS-CoV-2 Omicron Sublineages. *Nature* **2022**, *604*, 553–556, doi:10.1038/s41586-022-04594-4.
13. Leier, H.C.; Bates, T.A.; Lyski, Z.L.; McBride, S.K.; X. Lee, D.; Coulter, F.J.; Goodman, J.R.; Lu, Z.; Curlin, M.E.; Messer, W.B.; et al. Previously Infected Vaccinees Broadly Neutralize SARS-CoV-2 Variants. *medRxiv* **2021**, doi:10.1101/2021.04.25.21256049.
14. Liu, Y.; Liu, J.; Xia, H.; Zhang, X.; Fontes-Garfias, C.R.; Swanson, K.A.; Cai, H.; Sarkar, R.; Chen, W.; Cutler, M.; et al. Neutralizing Activity of BNT162b2-Elicited Serum. *N. Engl. J. Med.* **2021**, *384*, 1466–1468, doi:10.1056/NEJMc2102017.
15. Lu, L.; Mok, B.W.Y.; Chen, L.L.; Chan, J.M.C.; Tsang, O.T.Y.; Lam, B.H.S.; Chuang, V.W.M.; Chu, A.W.H.; Chan, W.M.; Ip, J.D.; et al. Neutralization of Severe Acute Respiratory Syndrome Coronavirus 2 Omicron Variant by Sera From BNT162b2 or CoronaVac Vaccine Recipients. *Clinical Infectious Diseases* **2022**, *75*, e822–e826, doi:10.1093/cid/ciab1041.
16. Lustig, Y.; Zuckerman, N.; Nemet, I.; Atari, N.; Kliker, L.; Regev-Yochay, G.; Sapir, E.; Mor, O.; Alroy-Preis, S.; Mendelson, E.; et al. Neutralising Capacity against Delta (B.1.617.2) and Other Variants of Concern Following Comirnaty (BNT162b2, BioNTech/Pfizer) Vaccination in Health Care Workers, Israel. *Eurosurveillance* **2021**, *26*, doi:10.2807/1560-7917.ES.2021.26.26.2100557.
17. Lyke, K.E.; Atmar, R.L.; Islas, C.D.; Posavad, C.M.; Szydlo, D.; Paul Chourdhury, R.; Deming, M.E.; Eaton, A.; Jackson, L.A.; Branche, A.R.; et al. Rapid Decline in Vaccine-Boosted Neutralizing Antibodies against SARS-CoV-2 Omicron Variant. *Cell Reports Medicine* **2022**, *3*, 100679, doi:10.1016/j.xcrm.2022.100679.

18. McCallum, M.; Bassi, J.; De Marco, A.; Chen, A.; Walls, A.C.; Di Iulio, J.; Tortorici, M.A.; Navarro, M.-J.; Silacci-Fregni, C.; Saliba, C.; et al. SARS-CoV-2 Immune Evasion by the B.1.427/B.1.429 Variant of Concern. *Science* **2021**, *373*, 648–654, doi:10.1126/science.abi7994.
19. Qu, P.; Faraone, J.N.; Evans, J.P.; Zheng, Y.-M.; Yu, L.; Ma, Q.; Carlin, C.; Lozanski, G.; Saif, L.J.; Oltz, E.M.; et al. Durability of Booster mRNA Vaccine against SARS-CoV-2 BA.2.12.1, BA.4, and BA.5 Subvariants. *N. Engl. J. Med.* **2022**, *387*, 1329–1331, doi:10.1056/NEJMc2210546.
20. Su, D.; Li, X.; He, C.; Huang, X.; Chen, M.; Wang, Q.; Qin, W.; Liang, Y.; Xu, R.; Wu, J.; et al. Broad Neutralization against SARS-CoV-2 Variants Induced by a Modified B.1.351 Protein-Based COVID-19 Vaccine Candidate. *bioRxiv* **2021**, 2021.05.16.444369, doi:10.1101/2021.05.16.444369.
21. Tang, J.; Lee, Y.; Ravichandran, S.; Grubbs, G.; Huang, C.; Stauft, C.; Wang, T.; Golding, B.; Golding, H.; Khurana, S. Reduced Neutralization of SARS-CoV-2 Variants by Convalescent Plasma and Hyperimmune Intravenous Immunoglobulins for Treatment of COVID-19. *bioRxiv* **2021**, 2021.03.19.436183, doi:10.1101/2021.03.19.436183.
22. van Gils, M.J.; Lavell, A.; van der Straten, K.; Appelman, B.; Bontjer, I.; Poniman, M.; Burger, J.A.; Oomen, M.; Bouhuijs, J.H.; van Vught, L.A.; et al. Antibody Responses against SARS-CoV-2 Variants Induced by Four Different SARS-CoV-2 Vaccines in Health Care Workers in the Netherlands: A Prospective Cohort Study. *PLOS Medicine* **2022**, *19*, e1003991, doi:10.1371/journal.pmed.1003991.
23. Wilhelm, A.; Widera, M.; Grikscheit, K.; Toptan, T.; Schenk, B.; Pallas, C.; Metzler, M.; Kohmer, N.; Hoehl, S.; Helfritz, F.A.; et al. Reduced Neutralization of SARS-CoV-2 Omicron Variant by Vaccine Sera and Monoclonal Antibodies. *medRxiv* **2021**, doi:10.1101/2021.12.07.21267432.
24. Wu, K.; Werner, A.P.; Moliva, J.I.; Koch, M.; Choi, A.; Stewart-Jones, G.B.E.; Bennett, H.; Boyoglu-Barnum, S.; Shi, W.; Graham, B.S.; et al. *mRNA-1273 Vaccine Induces Neutralizing Antibodies against Spike Mutants from Global SARS-CoV-2 Variants*; Immunology, 2021;
25. Yu, J.; Collier, A.Y.; Rowe, M.; Mardas, F.; Ventura, J.D.; Wan, H.; Miller, J.; Powers, O.; Chung, B.; Siamatu, M.; et al. Neutralization of the SARS-CoV-2 Omicron BA.1 and BA.2 Variants. *N. Engl. J. Med.* **2022**, *386*, 1579–1580, doi:10.1056/NEJMc2201849.
26. Yu, X.; Wei, D.; Xu, W.; Liu, C.; Guo, W.; Li, X.; Tan, W.; Liu, L.; Zhang, X.; Qu, J.; et al. Neutralizing Activity of BBIBP-CorV Vaccine-Elicited Sera against Beta, Delta and Other SARS-CoV-2 Variants of Concern. *Nat. Comm.* **2022**, *13*, 1788, doi:10.1038/s41467-022-29477-0.
27. Polinski, J.M.; Weckstein, A.R.; Batech, M.; Kabelac, C.; Kamath, T.; Harvey, R.; Jain, S.; Rassen, J.A.; Khan, N.; Schneeweiss, S. Durability of the Single-Dose Ad26.COV2.S Vaccine in the Prevention of COVID-19 Infections and Hospitalizations in the US Before and During the Delta Variant Surge. *JAMA Network Open* **2022**, *5*, e222959–e222959, doi:10.1001/jamanetworkopen.2022.2959.
28. Skowronski, D.M.; Setayeshgar, S.; Febriani, Y.; Ouakki, M.; Zou, M.; Talbot, D.; Prystajecky, N.; Tyson, J.R.; Gilca, R.; Brousseau, N.; et al. Two-Dose SARS-CoV-2 Vaccine Effectiveness with Mixed Schedules and Extended Dosing Intervals: Test-Negative Design Studies from British Columbia and Quebec, Canada. *medRxiv* **2021**, 2021.10.26.21265397, doi:10.1101/2021.10.26.21265397.

29. Andrews, N.; Tessier, E.; Stowe, J.; Gower, C.; Kirsebom, F.; Simmons, R.; Gallagher, E.; Chand, M.; Brown, K.; Ladhani, S.N.; et al. Vaccine Effectiveness and Duration of Protection of Comirnaty, Vaxzevria and Spikevax against Mild and Severe COVID-19 in the UK. *medRxiv* **2021**, doi:10.1101/2021.09.15.21263583.
30. Stowe, J.; Andrews, N.; Kirsebom, F.; Ramsay, M.; Bernal, J.L. Effectiveness of COVID-19 Vaccines against Omicron and Delta Hospitalisation: Test Negative Case-Control Study. *medRxiv* **2022**, doi:10.1101/2022.04.01.22273281.
31. Haas, E.J.; Angulo, F.J.; McLaughlin, J.M.; Anis, E.; Singer, S.R.; Khan, F.; Brooks, N.; Smaja, M.; Mircus, G.; Pan, K.; et al. Impact and Effectiveness of mRNA BNT162b2 Vaccine against SARS-CoV-2 Infections and COVID-19 Cases, Hospitalisations, and Deaths Following a Nationwide Vaccination Campaign in Israel: An Observational Study Using National Surveillance Data. *The Lancet* **2021**, *397*, 1819–1829, doi:10.1016/S0140-6736(21)00947-8.
32. Martínez-Baz, I.; Miqueleiz, A.; Casado, I.; Navascués, A.; Trobajo-Sanmartín, C.; Burgui, C.; Guevara, M.; Ezpeleta, C.; Castilla, J.; Working Group for the Study of COVID-19 in Navarra Effectiveness of COVID-19 Vaccines in Preventing SARS-CoV-2 Infection and Hospitalisation, Navarre, Spain, January to April 2021. *Eurosurveillance* **2021**, *26*, doi:10.2807/1560-7917.ES.2021.26.21.2100438.
33. Nasreen, S.; Chung, H.; He, S.; Brown, K.A.; Gubbay, J.B.; Buchan, S.A.; Fell, D.B.; Austin, P.C.; Schwartz, K.L.; Sundaram, M.E.; et al. Effectiveness of COVID-19 Vaccines against Symptomatic SARS-CoV-2 Infection and Severe Outcomes with Variants of Concern in Ontario. *Nat. Microbiol.* **2022**, *7*, 379–385, doi:10.1038/s41564-021-01053-0.
34. Bajema, K.L.; Dahl, R.M.; Prill, M.M.; Meites, E.; Rodriguez-Barradas, M.C.; Marconi, V.C.; Beenhouwer, D.O.; Brown, S.T.; Holodniy, M.; Lucero-Obusan, C.; et al. Effectiveness of COVID-19 mRNA Vaccines Against COVID-19–Associated Hospitalization — Five Veterans Affairs Medical Centers, United States, February 1–August 6, 2021. *MMWR Morb. Mortal. Wkly. Rep.* **2021**, *70*, 1294–1299, doi:10.15585/mmwr.mm7037e3.
35. Tartof, S.Y.; Slezak, J.M.; Fischer, H.; Hong, V.; Ackerson, B.K.; Ranasinghe, O.N.; Frankland, T.B.; Ogun, O.A.; Zamparo, J.M.; Gray, S.; et al. Effectiveness of mRNA BNT162b2 COVID-19 Vaccine up to 6 Months in a Large Integrated Health System in the USA: A Retrospective Cohort Study. *The Lancet* **2021**, S0140673621021838, doi:10.1016/S0140-6736(21)02183-8.
36. Bruxvoort, K.J.; Sy, L.S.; Qian, L.; Ackerson, B.K.; Luo, Y.; Lee, G.S.; Tian, Y.; Florea, A.; Aragones, M.; Tubert, J.E.; et al. Effectiveness of mRNA-1273 against Delta, Mu, and Other Emerging Variants. *medRxiv* **2021**, 2021.09.29.21264199, doi:10.1101/2021.09.29.21264199.
37. Ranzani, O.T.; dos Santos Leite, R.; Castilho, L.D.; Maymone Gonçalves, C.C.; Resende, G.; de Melo, R.L.; Croda, J. Vaccine Effectiveness of Ad26.COV2.S against Symptomatic COVID-19 and Clinical Outcomes in Brazil: A Test-Negative Study Design. *medRxiv* **2021**, 2021.10.15.21265006, doi:10.1101/2021.10.15.21265006.
38. Lopez Bernal, J.; Andrews, N.; Gower, C.; Gallagher, E.; Simmons, R.; Thelwall, S.; Stowe, J.; Tessier, E.; Groves, N.; Dabrera, G.; et al. Effectiveness of Covid-19 Vaccines against the B.1.617.2 (Delta) Variant. *N. Engl. J. Med.* **2021**, *385*, 585–594, doi:10.1056/NEJMoa2108891.

39. Heath, P.T.; Galiza, E.P.; Baxter, D.N.; Boffito, M.; Browne, D.; Burns, F.; Chadwick, D.R.; Clark, R.; Cosgrove, C.; Galloway, J.; et al. Safety and Efficacy of NVX-CoV2373 Covid-19 Vaccine. *N. Engl. J. Med.* **2021**, *385*, 1172–1183, doi:10.1056/NEJMoa2107659.
40. Emary, K.R.W.; Golubchik, T.; Aley, P.K.; Ariani, C.V.; Angus, B.J.; Bibi, S.; Blane, B.; Bonsall, D.; Cicconi, P.; Charlton, S.; et al. Efficacy of ChAdOx1 nCoV-19 (AZD1222) Vaccine against SARS-CoV-2 VOC 202012/01 (B.1.1.7). *SSRN Journal* **2021**, doi:10.2139/ssrn.3779160.
41. Sadoff, J.; Gray, G.; Vandebosch, A.; Cárdenas, V.; Shukarev, G.; Grinsztejn, B.; Goepfert, P.A.; Truyers, C.; Fennema, H.; Spiessens, B.; et al. Safety and Efficacy of Single-Dose Ad26.COV2.S Vaccine against Covid-19. *N. Engl. J. Med.* **2021**, *384*, 2187–2201, doi:10.1056/NEJMoa2101544.
42. Shinde, V.; Bhikha, S.; Hoosain, Z.; Archary, M.; Bhorat, Q.; Fairlie, L.; Lalloo, U.; Masilela, M.S.L.; Moodley, D.; Hanley, S.; et al. Efficacy of NVX-CoV2373 Covid-19 Vaccine against the B.1.351 Variant. *N. Engl. J. Med.* **2021**, *384*, 1899–1909, doi:10.1056/NEJMoa2103055.
43. Al Kaabi, N.; Zhang, Y.; Xia, S.; Yang, Y.; Al Qahtani, M.M.; Abdulrazzaq, N.; Al Nusair, M.; Hassany, M.; Jawad, J.S.; Abdalla, J.; et al. Effect of 2 Inactivated SARS-CoV-2 Vaccines on Symptomatic COVID-19 Infection in Adults: A Randomized Clinical Trial. *JAMA* **2021**, *326*, 35, doi:10.1001/jama.2021.8565.
44. Voysey, M.; Clemens, S.A.C.; Madhi, S.A.; Weckx, L.Y.; Folegatti, P.M.; Aley, P.K.; Angus, B.; Baillie, V.L.; Barnabas, S.L.; Bhorat, Q.E.; et al. Safety and Efficacy of the ChAdOx1 nCoV-19 Vaccine (AZD1222) against SARS-CoV-2: An Interim Analysis of Four Randomised Controlled Trials in Brazil, South Africa, and the UK. *The Lancet* **2021**, *397*, 99–111, doi:10.1016/S0140-6736(20)32661-1.
45. Tanriover, M.D.; Doğanay, H.L.; Akova, M.; Güner, H.R.; Azap, A.; Akhan, S.; Köse, Ş.; Erdinç, F.Ş.; Akalın, E.H.; Tabak, Ö.F.; et al. Efficacy and Safety of an Inactivated Whole-Virion SARS-CoV-2 Vaccine (CoronaVac): Interim Results of a Double-Blind, Randomised, Placebo-Controlled, Phase 3 Trial in Turkey. *The Lancet* **2021**, *398*, 213–222, doi:10.1016/S0140-6736(21)01429-X.
46. Jakarta, W.S.I.C.I. Indonesia Grants Emergency Approval for Sinovac Vaccine. *The Straits Times* 2021.
47. Baden, L.R.; El Sahly, H.M.; Essink, B.; Kotloff, K.; Frey, S.; Novak, R.; Diemert, D.; Spector, S.A.; Rouphael, N.; Creech, C.B.; et al. Efficacy and Safety of the mRNA-1273 SARS-CoV-2 Vaccine. *N. Engl. J. Med.* **2021**, *384*, 403–416, doi:10.1056/NEJMoa2035389.
48. Novavax COVID-19 Vaccine Demonstrates 90% Overall Efficacy and 100% Protection Against Moderate and Severe Disease in PREVENT-19 Phase 3 Trial Available online: https://ir.novavax.com/2021-06-14-Novavax-COVID-19-Vaccine-Demonstrates-90-Overall-Efficacy-and-100-Protection-Against-Moderate-and-Severe-Disease-in-PREVENT-19-Phase-3-Trial (accessed on 30 March 2023).
49. Polack, F.P.; Thomas, S.J.; Kitchin, N.; Absalon, J.; Gurtman, A.; Lockhart, S.; Perez, J.L.; Pérez Marc, G.; Moreira, E.D.; Zerbini, C.; et al. Safety and Efficacy of the BNT162b2 mRNA Covid-19 Vaccine. *N. Engl. J. Med.* **2020**, *383*, 2603–2615, doi:10.1056/NEJMoa2034577.

50. Logunov, D.Y.; Dolzhikova, I.V.; Shcheblyakov, D.V.; Tukhvatulin, A.I.; Zubkova, O.V.; Dzharullaeva, A.S.; Kovyrshina, A.V.; Lubenets, N.L.; Grousova, D.M.; Erokhova, A.S.; et al. Safety and Efficacy of an rAd26 and rAd5 Vector-Based Heterologous Prime-Boost COVID-19 Vaccine: An Interim Analysis of a Randomised Controlled Phase 3 Trial in Russia. *The Lancet* **2021**, *397*, 671–681, doi:10.1016/S0140-6736(21)00234-8.
51. Tang, P.; Hasan, M.R.; Chemaitelly, H.; Yassine, H.M.; Benslimane, F.M.; Al Khatib, H.A.; AlMukdad, S.; Coyle, P.; Ayoub, H.H.; Al Kanaani, Z.; et al. BNT162b2 and mRNA-1273 COVID-19 Vaccine Effectiveness against the SARS-CoV-2 Delta Variant in Qatar. *Nat. Med.* **2021**, *27*, 2136–2143, doi:10.1038/s41591-021-01583-4.
52. Nordström, P.; Ballin, M.; Nordström, A. Effectiveness of Heterologous ChAdOx1 nCoV-19 and mRNA Prime-Boost Vaccination against Symptomatic Covid-19 Infection in Sweden: A Nationwide Cohort Study. *The Lancet Regional Health - Europe* **2021**, *11*, 100249, doi:10.1016/j.lanepe.2021.100249.
53. Tseng, H.F.; Ackerson, B.K.; Luo, Y.; Sy, L.S.; Talarico, C.A.; Tian, Y.; Bruxvoort, K.J.; Tubert, J.E.; Florea, A.; Ku, J.H.; et al. Effectiveness of mRNA-1273 against SARS-CoV-2 Omicron and Delta Variants. *Nat. Med.* **2022**, *28*, 1063–1071, doi:10.1038/s41591-022-01753-y.
54. Hansen, C.; Schelde, A.; Moustsen-Helm, I.; Embor, H.-D.; Eriksen, R.; Stegger, M.; Krause, T.; Mølbak, K.; Valentiner-Branth, P. Vaccine Effectiveness against Infection and COVID-19-Associated Hospitalisation with the Omicron (B.1.1.529) Variant after Vaccination with the BNT162b2 or mRNA-1273 Vaccine: A Nationwide Danish Cohort Study. *Research Square* **2022**, doi:10.21203/rs.3.rs-1486018/v1.
55. *SARS-CoV-2 Variants of Concern and Variants under Investigation*; UK Health Security Agency, 2021;
56. Andrews, N.; Stowe, J.; Kirsebom, F.; Toffa, S.; Rickeard, T.; Gallagher, E.; Gower, C.; Kall, M.; Groves, N.; O'Connell, A.-M.; et al. Covid-19 Vaccine Effectiveness against the Omicron (B.1.1.529) Variant. *N .Engl. J. Med.* **2022**, *386*, 1532–1546, doi:10.1056/NEJMoa2119451.
